# Avenues of decarbonisation in the dynamics of processed food supply chains: Towards responsible production consumption

**DOI:** 10.1016/j.heliyon.2024.e26456

**Published:** 2024-02-17

**Authors:** Janpriy Sharma, Shweta Singh, Mohit Tyagi, Satvasheel Powar

**Affiliations:** a Department of Information Engineering and Computer Science, University of Trento, Povo, 38123, Italy; b Bureau of Economic Geology, Jackson School of Geosciences, The University of Texas at Austin, Texas, 78758, USA; c Department of Production and Industrial Engineering, Punjab Engineering College, Chandigarh, 160012, India; d School of Mechanical and Materials Engineering, Indian Institute of Technology, Mandi, Himachal Pradesh, 175005, India; e School of Technology and Business Studies, Energy Technology, Högskolan Dalarna, Borlänge 78170, Sweden

**Keywords:** Sustainable food supply chains, Food transformation, Decarbonisation, Environmental impact, Hesitant fuzzy sets, Entropy measure

## Abstract

Nowadays, the demand for processed food items is surging. To fulfil the enhanced demand, a significant impact is laid on the environment, which enhances the carbon footprint being generated. Hence, to overcome this, the avenues of decarbonisation need to be explored. The presented work is aimed at promoting the decarbonisation of the existing practices within the processed food supply chains. It finds strong compliance with the sustainable development goal (SDG-12), focusing on responsible production-consumption mechanisms. For the same, key enactors of decarbonisation are identified and mapped with the practices at various stages of food supply chains, i.e. upstream, downstream, and other allied practices. Based upon these enactors, a relational, hierarchical framework is developed to provide a comprehensive perspective on complex intricacies. This framework is analysed with an innovative approach which comprises the fundamentals of Interval-Valued Intuitionistic Hesitant Fuzzy Set with the Entropy measures. It results in the outranking of the enactors relative to its importance in the decarbonisation of processed food supply chains. Furthermore, the empirical findings are validated by the sensitivity analysis to felicitate robust decision-making. The outcomes of the presented work provide a roadmap and stepped approach to achieve the decarbonisation goals and make production-consumption mechanisms sustainable. It finds implications in the development of the framework, policy formulation, and decisional attributes for the decarbonisation of food supply chains. It focuses on the adoption of strategies that align with global efforts to mitigate climate change and promote a sustainable future.

## List of Abbreviation

DM: Decision Makers
FSC: Food Supply Chain
FSSAI: Food Safety and Standards Authority of India
GHI: Global Hunger Index
IVIFS: Interval Valued Intuitionistic Fuzzy Set
IVIHFS: Interval-Valued Intuitionistic Hesitant Fuzzy Set
SDG: Sustainable Development Goals
SFSC: Sustainability Food Supply Chain
UN: United Nation

## 1 Introduction

The demand for various food items has increased threefold due to the exponential increase in the world's population from 2.53 billion in 1950 to 7.9 billion in November 2021 [1]. Human consumption has also increased by 30% compared to nature's production capacity [2]. The population's steady growth has also increased the need for nurturing and feeding the masses. Furthermore, according to the GHI Index 2020, the sustainable development goal of zero hunger by 2030 is difficult to achieve, and 37 countries are expected to fall short of even reaching low hunger by 2030 [3]. Africa and South Asia are among the regions with the highest levels of hunger and malnutrition [4].

**Fig. 1:**
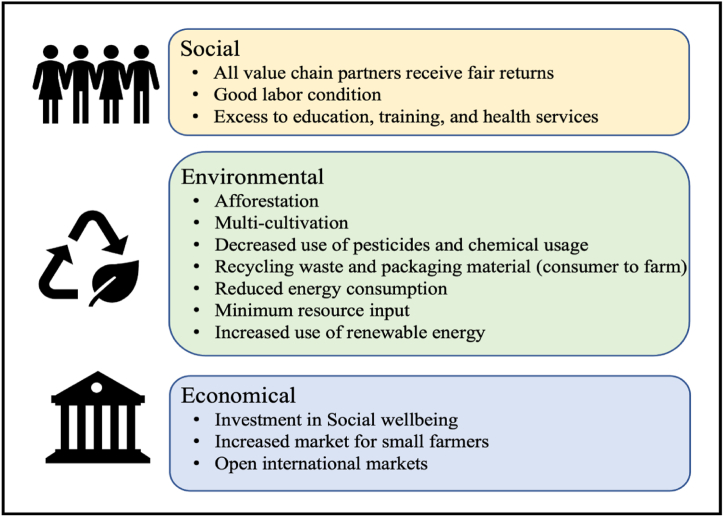
Dimensions of sustainability in food supply chain.

Focusing on the country of interest, India ranks second in global farm exports, first in net cropped area, and high in production of various food categories. Despite numerous achievements in the food industry, the Food Safety and Standards Authority of India (FSSAI) has estimated an annual loss of approximately 6 billion USD due to food waste [5]. The reason is that Food Supply Chains (FSCs) and their intermediaries have been disrupted. Other factors contributing to food business losses and supply chain inefficiencies include a lack of demand-supply drivers, improper packing and manual handling, poor infrastructure and cold chain infrastructure, a lack of technological adoption, and so on. As a result, Food Supply Chains (FSCs) must provide new and improved structures to meet consumer demands and improve the efficiency of the food sector. However, FSCs alone are not enough; sustainability practices in various stages of FSCs (from farm to fork) are important. Sustainable Food Supply Chain (SFSC) maps the supply and demand pattern by underpinning the social, economic and environmental perspectives [[6], [7]]. It is important to establish SFSCs as the dynamics of conventional FSCs put the environmental, economic, and social stakes at fire, blurring the future avenues of upcoming generations. In this direction, the United Nations (UN) also, in the year 2015, defined the 12th sustainable development goals (SDGs) for “sustainable production and consumption practices” [8]. Some essential indicators of including sustainability practices in FSCs are shown in Fig. 1. It depicts the dimension of sustainability associated with the food supply chin dynamics, under the pillar of social, economy and environment.

In India, the majority of the food processing industries are small and medium-sized, and they are not well-versed in sustainable practices [9]. As a result, there is a need to bridge the gap between existing FSC practices and long-term goals [10]. Among the three sustainability pillars, economic and social indicators are examined in the majority of papers for FSCs, but the key indicator that played the main role here (in FSCs) is environmental indicators, which are examined only marginally despite their enormous effects.

Beginning with agricultural activities, which include land conversion, the use of various fertilizers, livestock rearing, and its associated wastes, the FSC in transition from farm to fork accounts for nearly 18% of global greenhouse emissions and almost 80% of land usage [11]. Agriculture activities feed a variety of food processing industries, which include a variety of high-energy-consuming processes such as cutting, mixing, forming, extraction, fermentation, dehydration, chilling, boiling, and so on, depending on the nature of the product. All of these processes produce massive carbon footprints. More than 40% of food items require refrigeration, which consumes nearly 15% of global electricity production [12]. Furthermore, if a product includes baking procedures, it consumes a large amount of energy, as it implies equipment with high electrical loads. The environment will be burdened with a variety of greenhouse emissions and associated carbon deposits. Activities related to retailing and distribution via a network of supermarkets, grocery stores, and restaurants add to the carbon footprint [13]. Aside from that, some activities, such as food preservation and displaying perishable and non-perishable food products on shelves/counters for sale, contribute significantly to carbon footprints due to the extensive use of energy by heating and cooling equipment, ambient lighting, and so on [14]. It is estimated that the dynamics of the FSC contribute 31% of greenhouse gases to the environment and 50% of eutrophication [15].

In addition, research has shown that the transportation phase of food supply chains is the most difficult [16]. Regardless of how carefully items are packaged and maintained to avoid contamination, there is always an expense associated with the long-distance transportation and storage of food [17]. The price is typically paid by causing problems for the two most important indicators of sustainability, which are the economic (high prices for petroleum) and environmental systems (massive carbon footprint). When the energy consumption in the FSC of developed nations and developing nations is compared, it is estimated that developed countries use approximately 48% of the total energy in food processing and distribution. In comparison, about 43% of the total energy in developing nations is used in the cooking and preparation of food for consumption purposes. This difference in energy utilization can be attributed to the fact that developed countries have more sophisticated and energy-seeking food processing and distribution systems [18]. For instance, in Canada, the import of food items accounts for 61 billion ton-kilometres of transverse distance, which results in 3.3 million Mt of emissions from carbon-based sources [19]. Furthermore, in the United Kingdom, annual food consumption transverses nearly 30 billion vehicle kilometres, of which almost 25% of the petroleum product is consumed in related logistics practices, producing a massive carbon footprint counting roughly 10% of the total [12, 20].

Food industries are working restlessly to overcome the food safety, public health, and cost-effective availability of commodities. But nowadays, there is a makeshift towards the social, economic, and environmental perspectives. It is aimed to decarbonise the existing food supply chain dynamics. Hence, a need arises to develop a framework which could expedite carbon neutrality in food supply chains. Owing to same presented work is grounded to answer the Research Questions (*RQs)*.

RQ1 What are the various critical enactors of decarbonisation at the different operational tiers of the processed FSC performance system?

RQ2 What is the interrelationship between the FSC practices and the enactors of decarbonisation of FSCs?

RQ3 How to implement the developed framework of decarbonisation of processed FSC performance system and felicitate decision making process?

### 1.1 Work contribution

This work contributes to the domain and practice by developing a robust framework which clusters the various enactors of the decarbonisation of the processed food supply chain at its different operational tiers distinctly. Shabir et al. [21] assessed the carbon footprint being generated at various levels of food supply chain operations. Cited the need for a comprehensive framework to decarbonise the existing food supply chain performance system and improve sustainability. Kumar et al. [22] detailed the requirement of a holistic roadmap aimed at decarbonising the developing economies' supply chain operations. Xu et al. [23] analysed the need for a transition of the existing food supply chain network towards the decarbonisation avenues. Mishra et al. [24] insisted on the need for critical enactors which can felicitate the decision-making in supply chain operations for achieving carbon neutrality. Ada et al. [25] reviewed the current food supply chain and cited the development of a framework to combat the carbon footprint being generated.

This work leaps over the existing studies by identifying the key enactors of decarbonisation and clustering them within the various stages of food supply chain (FSC) operations. It uniquely attributes every practice of FSCs with enactors of decarbonisation, yielding towards the development of a comprehensive framework. Furthermore, to enrich the synthesis and decision-making process, it outranks enactors owing to its vitality in promoting decarbonisation within operations, strategically as well as tactically. In order to capture the field practicalities precisely, enactors are analysed with the methodology of Entropy measure, which is enveloped with an Interval-Valued Intuitionistic Hesitant Fuzzy Set. It captures the critics of decisional and operational uncertainties, which regulates the strength of the policy-making process and guides a smooth transition towards decarbonisation avenues.

### 1.2 Research roadmap

**Fig. 2:**
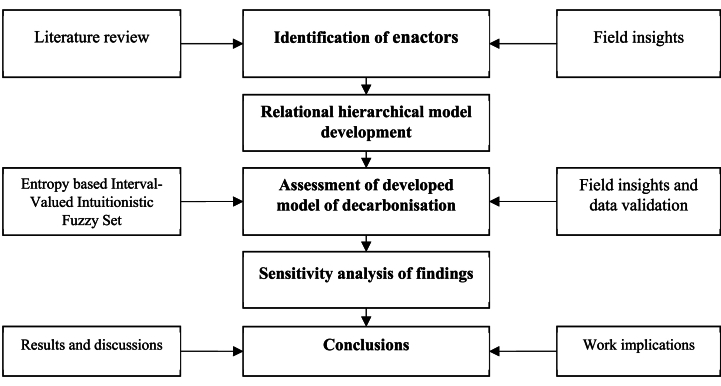
Research roadmap of the presented work.

The presented work opts for the research roadmap, which is detailed in Fig. 2, for better visualization. It begins with the identification of enactors, which results in development of relation hierarchical model. The developed model is assessed the methodology measuring entropy of enactors by integrating fundamental of interval valued hesitant fuzzy set theory.

India is the third largest emitter of 2.9 gigatons of carbon emissions globally. Hence, the need arises to prepare a roadmap towards the decarbonised perspective in India for an orderly transition. As a part of the sustainability prospects, concerned bodies and organisations are focusing massively on decarbonisation goals to secure avenues of net-zero carbon emission by 2050. Evidently, global food production accounts for 80 per cent of global deforestation and 29 per cent of greenhouse emissions [26]. Furthermore, the social perspective bundled with the food supply chain also needs to be accounted for, as 25–50 percent of food produced for consumption gets wasted. Hence, it becomes vital to steer the existing food supply chain dynamics towards decarbonisation practices.

## 2 Literature review

Nowadays, the rampant exponential growth of the industries and surging population count have burdened the FSCs with the enhanced volume of the carbon footprint. Furthermore, bridging the gap between the soaring demand and its associated supply patterns of processed food items has carved instances favouring the inclusion of sustainability perspectives in the FSC dynamics. In continuation of the same, Goggins [27] focused on the various important contextual factors that influence the provision of agri-food across organisations. Furthermore, the study identified the various opportunities favouring improvement of sustainability within FSC dynamics and recommending a sustainable food strategy. It implied the theoretical working approach and remained constrained to procurement practices. Morley [28] explored the impact of sustainable agri-food demands on the firms along with the innovative potential bundled with sustainable procurement in FSCs. This study focused on the public procurement strategies in the United Kingdom as a key enabler of decarbonisation in food processing and distribution. It incorporated the survey-based outcomes, which are theoretically explained but focused only on the procurement protocols. Gonzalez-Diaz et al. [29] analysed the various critics of the rice and wheat supply chain towards the avenues of decarbonisation. For the same, a life cycle assessment is done to estimate the levels of the carbon footprint being generated during the processing of rice and wheat. The outcomes of the study focused on the peripherals of the processing in the supply chain and did not bind with the holistic working approach. Li et al. [30] studied the various low-carbon strategies by considering the perspective of manufacturers in FSC dynamics. This study details the cap and trade mechanisms, which are further investigated by the game theory-based working approach. The outcomes of the study focused on the technological advancements that can endure the manufacturing in FSC towards decarbonisation. Attari et al. [31] comprehensively reviewed the electric power supply chain network and focused on multiple perspective associated with costing, emission and operations strategy. Ala et al. [32] developed a bi-objective decision model to ensure long-term resilience in sustainable energy planning. Developed a strategic energy roadmap based upon the neutrosophic-based multi-objective grey wolf optimisation.

Acquaye et al. [33] presented the analytical view of agri-food supply chains and the effects of the carbon footprint being generated by considering multi-regional insights. It focused on the development of a robust environmental sustainability performance measurement model. Outcomes revealed the evaluation of the total carbon footprint being generated over the considered timeline of twenty years. This study remained constrained up to the evaluation of the carbon footprint being generated but did not deliberate the decarbonisation perspectives. Wickham et al. [34] considered the tier of distribution and retailing in FSC and assessed the carbon footprint being generated in its dynamics. This study considered the insights of British industries and outcomes evaluated the optimal cost configurations. However, its outcomes remained specific to the logistics in the dynamics of FSC without considering its holistic perspective. Kolahi-Randji et al. [35] implied simulation based approach to assess the impact of different strategies on profit enhancement across the supply chain tiers. Rootzén et al. [36] analysed the collaborative actions of the supply chain aiming for zero carbon emission. For the same, various barriers and prevailing opportunities are detailed by focusing on the theoretical perspectives. Bataille et al. [37] modelled the supply chains for zero-emission and grounded the policies focusing on its mitigation. This study emphasised the theoretical throughputs in the supply chain, the circumference of the development of a mitigation-based framework and the sequencing of the industrial transformations. Sharma et al. [38] focused on the perspective of IoT based system deployment in FSC to overcome the operational inefficiencies and improve sustainability. Peterson et al. [39] explored the market portfolios of FSC and surveyed the consumer choices for sustainability and decarbonisation. The outcomes of this study focused on the development of a sustainable marketing framework by implying statistical analysis. Ala et al. [32] developed a fuzzy optimisation based model for handling the uncertainties involved in supply chain network design aimed to provide sustainable health care. Bellemare et al. [40] focused on the various perspectives of consumers in agri-food-based value chains in low and middle-income countries.

### 2.1 Research gaps

Pearson et al. [41] reviewed the current food supply chain performance system and focused on the requirement identification of key factors contributing in decarbonisation. Burgess et al. [42] focused on the requisition of transforming the existing food supply chain practices towards decarbonisation. Sovacool et al. [11] cited the need of framework governing the decarbonisation perspectives for sustainable prospects in FSCs for developing economies. Kassahun et al. [43] revealed the need of understanding the interlinkage between the decarbonisation and sustainability in FSC dynamics. Furthermore, securing decarbonisation in processed FSC, safeguards environment, leverages economic potencies and societal benefits [44]. Yadav et al. [45] developed a conceptual framework for sustainable food supply chain operations and suggested the need for the development of key performance indicators to make FSC future ready for sustainable future needs. Agnusdei and Coluccia [46] analysed the need of comprehensive perspective of FSC performance system from farm to fork, accumulating the insights of sustainable production-consumption. Tola et al. [47] focused on balancing the food production system output and curtail the carbon-footprint being generated. Detailed the need of understanding of the FSC dynamics and possible sustainable actions at its every working tier*.* Hence, promoting decarbonisation carves the way towards the sustainable avenues.

An inference can be grounded that need arises to identify various key enactors which can mediate the existing food supply chain dynamics towards the decarbonisation. Furthermore, a framework is required to bridge the gap between the existing and decarbonised food supply chain performance system. Owing to this work identifies the key enactors of decarbonisation at various levels of FSCs and analyses them with the methodology of Entropy based Interval-Valued Intuitionistic Hesitant Fuzzy Set.

This research aims to fill these gaps through a holistic approach that incorporates the various FSC activities (from farm to folk) as well as sustainability factors. By incorporating sustainability into the dynamics of Indian processed FSC, this study's scientific contribution is seen in improving the economy, protecting the environment, and effectively nurturing society. This research identified its uniqueness in promoting sustainable practices in Indian FSC performance.

### 2.2 Model development

**Fig. 3:**
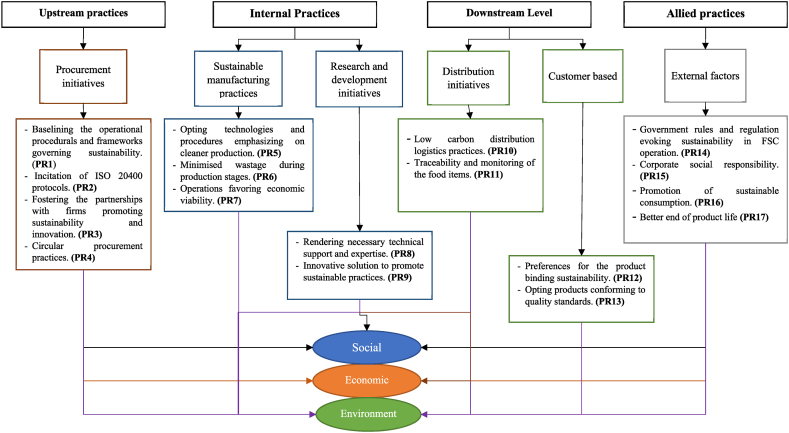
Developed model of the decarbonisation enactors in the FSC.

The presented work overviews the holistic perspective of the FSCs towards sustainable avenues and decarbonizing their dynamics. For the same, a relation hierarchical model is developed, seeding insights from the research literature. This model comprises the various tiers of FSCs and their associated enactors, focusing on decarbonisation perspectives. The details of the same are rendered in Table 1. Here, the notation *‘Hi’* (where *i* = 1,2 …,6) depicts the allied tier of the FSC, whereas *‘PRj’* (where *j* = *1,2, 3 ….,17)* indicates its associated practice. One sight of the developed model is shown in Fig. 3.

**Table 1:** Enactors of the decarbonisation in FSCPS.

Tier	Practice	Reference
Procurement initiatives **(H1)**	Baselining the operational procedurals and frameworks governing sustainability **(PR1)**	Ögel et al. [48]; Olan et al. [49]; Fraser et al. [50]
	Incitation of ISO 20400 protocols. **(PR2)**	Blind and Heß [51]; Zhao et al. [52]; Sharma et al. [53]
	Fostering partnerships with firms promoting sustainability and innovation. **(PR3)**	Thao et al. [54]; Zaccone et al. [55]; Grabs and Caroden [56]
	Circular procurement practices. **(PR4)**	Santeramo [44]; Capelleveen et al. [57]; Kristensen et al. [58]
Sustainable manufacturing practices **(H2)**	Opting technologies and procedures emphasizing cleaner production.**(PR5)**	De Bernardi et al. [59]; Chopra et al. [60]; Krishnan et al. [15]; Bappy et al. [61]
	Minimised wastage during production stages. **(PR6)**	Sharma et al. [62]; Almuflih et al. [63]; Mangla et al. [64]
	Operations favouring economic viability. **(PR7)**	Shabir et al. [21]; Sharma and Tyagi [65]; Dora et al. [66]
Research and development initiatives **(H3)**	Rendering necessary technical support and expertise. **(PR8)**	Dewi et al. [67]; Kumar et al. [68]; Jabbour et al. [69]; Tsolakis et al. [70]
	An innovative solution to promote sustainable practices. **(PR9)**	Joshi et al. [71]; Mahroof et al. [72]; Martin-Rios et al. [73]; Gupta et al. [74]
Distribution initiatives **(H4)**	Low carbon distribution logistics practices. **(PR10)**	Yang and Xu [75]; Tavassoli et al. [76]; Kashyap and Agarwal [77]
	Traceability and monitoring of the food items. **(PR11)**	Zhou et al. [78]; Lawo et al. [79]; Sharma and Jayant [80]; Kamilaris et al. [81]
Customer-based **(H5)**	Preferences for the product binding sustainability. **(PR12)**	Tripathi and Agarwal [82]; Sharma et al. [83]; Savarese et al. [84]
	Opting products conforming to quality standards. **(PR13)**	Costa et al. [85]; Sharma et al. [86]; Starobin [87]; Mardani et al. [88]
External factors **(H6)**	Government rules and regulations evoking sustainability in FSC operation. **(PR14)**	Lu et al. [89]; Sharma et al. [90]; Zhou and Xu [91]
	Corporate social responsibility. **(PR15)**	Sandberg et al. [92]; Kumar et al. [93]; Bubicz et al. [94]; Tyagi et al. [95]
	Promotion of sustainable consumption. **(PR16)**	Huang et al. [96]; Qureshi et al. [97]; Sharma et al. [98]; Dupouy and Gurinovic [99]
	Better end of product life. **(PR17)**	Yontar [100]; Stillitano et al. [101]; Singh and Tayal [102]; Dubey et al. [103]

Fig. 2 details the developed relational hierarchical model which comprises of the enactors of decarbonisation, mapped with the event of occurrence in food supply chain. All the enactors have strong compliances with the social, economic and environment concerns binding it to sustainable prosects.

## 3 Methodology

The entropy-based method, which is one of the underlying principles of IVIHFS, is used to investigate the proposed relational hierarchical model of sustainability. The model encompasses the various practices and initiatives associated with the FSC performance system in general. The goal is to quantify the uncertainty and hesitancy associated with decisional entity assessments and sustainability enactor assessments in processed FSC.

### 3.1 Why interval valued intuitionistic hesitant fuzzy set (IVIHFS)?

It is common that in human judgement, uncertainty and impreciseness often occur, which impacts the decision-making process. Hence, in order to handle the same, Zadeh proposed the fuzzy set theory, which can be incorporated into the decision-making process. It assigns membership function value that relates to the degree of uncertainty associated with human judgement [104]. In many real-time situations, judgment is bundled with a degree of hesitancy, which needs to be incorporated into decision-making. For the same, Torra [105] introduced the Hesitant fuzzy sets, which take into account the degree of hesitancy in decision-making along with the uncertainty. Hesitancy outlays the notions of lack of data, knowledge, information, and attention associated with the decision makers' perception. Advancing, in the domain to broaden the spectrum of hesitancy in decision making, interval-valued intuitionistic hesitant fuzzy sets (IVIHFS) are proposed.

The interval-valued intuitionistic hesitant fuzzy set (IVIHFS) comprises the membership and non-membership function value allied with hesitancy in decision-making. This broadens the range of membership as well as non-membership function values associated with the degree of hesitation in decision-making. For a better interpretation of the comparison between the existing fuzzy set theory extension and IVIHFS Table 2 is developed.

**Table 2:** Comparison of proposed fuzzy set theory with existing fuzzy theories.

	Membership function	Hesitancy Membership Function	Hesitant Membership and Non- Membership Function	Inference
**Fuzzy**	Yes	No	No	No Hesitancy incorporated in decision making.
**Hesitant Fuzzy Set**	Yes	Yes	No	Hesitancy incorporated in decision making.
**Interval Valued Intuitionistic Hesitant Fuzzy Set***(Proposed)*	Yes	Yes	Yes	Broad range of hesitancy in terms of membership and non-membership incorporated in decision making.

### 3.2 Some preliminaries of interval valued intuitionistic hesitant fuzzy set

Definition 1 Assume ‘*Z*’ as a reference set. A typical HFS depicted by ‘B’ on ‘*Z*’ is underpinned by function ${\prime k}_{B}{\left(z\right)}^{\prime }$ returning sub-set ranging [0,1]. Its mathematical interpretation is as shown in equation (1):

(1) $$B=\left\{\left\langle z,k_{B}\left(z\right)\right\rangle \;|z\in Z\right\}$$

Where, ${\prime k}_{B}{\left(z\right)}^{\prime }$ indicates the possible membership degree lying between 0 and 1 for elements z∈Z, allied with the set ‘B’*.* ${\prime k}_{B}{\left(z\right)}^{\prime }$ is recognized as the hesitant fuzzy element.

Definition 2 Torra [105] defined the empty and full HFS. For the considered HFS ‘*B*’, if k(z)={0} for all the z∈Z, then ‘B’ is referred to as null HFS (φ). Similarly, if k(z)={1} for z∈Z, it is said to be full HFS.

Definition 3 For the considered two HFS ‘*P*’ and ‘*Q*’ respectively, where $P=\left\{\left\langle z,k_{P}\left(z\right)\right\rangle \;|z\in Z\right\}$ and $Q=\left\langle z,k_{Q}\left(z\right)\right\rangle \;|z\in Z$. HFS ‘P’ is said to be a subset of ‘Q’ if sufficient condition of $h_{P}^{\theta \left(j\right)}\left(z\right)\leq h_{Q}^{\theta \left(j\right)}\left(z\right),\text{for}\;z\in Z$ is being satisfied.

Definition 4 Assumed HFS ‘P’ and ‘*Q*’, are said to be equal when the condition rendered in equation (2) is satisfied.

(2) If,P⊆QandP⊇Qthen,P=Q

Some of the allied operations of the HFS for the considered three hesitant fuzzy elements $k,k_{1},k_{2}$ respectively (Refer equations (3), (4), (5), (6), (7))).

(3) $$k^{c}=\underset{\delta \epsilon k}{⋃}\left\{1-\delta \right\}$$

(4) $$k_{1}\cup k_{2}=\left\{k\;\epsilon \;\left(k_{1}\cup k_{2}\right)|k\geq \max\;(k_{1}^{-},k_{2}^{+})\right\}\text{;}$$

(5) $$k_{1}\cap k_{2}=\left\{k\;\epsilon \;\left(k_{1}\cup k_{2}\right)|k\geq \min\;(k_{1}^{-},k_{2}^{+})\right\}\text{;}$$

(6) $$k_{1}⊕k_{2}=\underset{\theta \epsilon k_{1},k_{2}}{⋃}\left\{\theta_{1}+\theta_{1}-\theta_{1}\theta_{2}\right\}\text{;}$$

(7) $$k_{1}⨂k_{2}=\underset{\theta \epsilon k_{1},k_{2}}{⋃}\left\{\theta_{1}\theta_{2}\right\}\text{.}$$

Where, $k^{-}\left(z\right)=\min\;k\left(z\right)\;\text{and}\;k^{+}\left(z\right)=\max\;k\left(z\right)$ depicts the lower and upper bounds allied with the hesitant fuzzy elements.

Definition 5 Assume ‘D’ as an intuitionistic HFS in ‘Z' as universal discourse, comprising of the ‘m(z)’ and ‘n(z)’ as two functions ranging between 0 and 1. It can be interpreted mathematically as in equation (8).

(8) D={⟨z,m(z),n(z)⟩∀z∈Z}

where, m*(*z*)* and n*(*z*)* refers to the possible degree of the membership as well as non-membership function values, following the conditions as (Refer equation (9))

$$\beta \geq 0,\gamma \leq 1,0\leq \beta^{+}+\gamma^{+}\leq 1\;\forall \;\beta \;\epsilon \;m\left(z\right),\gamma \epsilon \;n\left(z\right)$$

(9) $$where\;\beta^{+}\;\epsilon \;m\left(z\right)=\underset{\beta \epsilon h\left(z\right)}{⋃}\max\left\{\beta \right\}\;\text{and}\;\gamma^{+}\;\epsilon \;n\left(z\right)=\underset{\gamma \epsilon h\left(z\right)}{⋃}\max\left\{\gamma \right\}\forall \;z\in Z$$

Definition 6 For the assumed ‘Z’ as the universe of discourse, ‘*E*’ is defined as IVIHFS as the function which returns the subset allied with the set of all possible IVIFS, within ‘Z’*.* The mathematical depiction of the IVIHFS is showcased in equation (10).

(10) $$E=\left\{\left\langle z,p_{E}\left(z\right)\right\rangle |z\;\epsilon \;Z\right\}$$

Where, $p_{E}\left(z\right)$ refers to the sets of some IVIFS, depicting the possible course of membership and non-membership degree-based intervals of elements in zϵZ. If say, $\alpha \;\epsilon \;p_{E}\left(z\right)$ then ‘α’ is said to be an IVIFS and depicted as $\alpha =\left(\mu_{\alpha },\vartheta_{\alpha }\right)$ = $\left(\left[\mu_{\alpha }^{-},\mu_{\alpha }^{+}\right],\left[\vartheta_{\alpha }^{-},\vartheta_{\alpha }^{+}\right]\right)$.

Definition 7 Let $G=\left\langle z_{i},\left(\left[\mu_{G}^{-}\left(z_{i}\right),\mu_{G}^{+}\left(z_{i}\right)\right],\left[\vartheta_{G}^{-}\left(z_{i}\right),\vartheta_{G}^{+}\left(z_{i}\right)\right]\right)\right\rangle$ and $H=\left\langle z_{i},\left(\left[\mu_{H}^{-}\left(z_{i}\right),\mu_{H}^{+}\left(z_{i}\right)\right],\left[\vartheta_{H}^{-}\left(z_{i}\right),\vartheta_{H}^{+}\left(z_{i}\right)\right]\right)\right\rangle$ as two IVIHFS on ‘Z’. its allied operation comprising of inclusion, complement, and equality respectively.

I. G⊆H if it satisfies the conditions $\mu_{G}^{-}\left(z_{i}\right)\leq \mu_{H}^{-}\left(z_{i}\right),\mu_{G}^{+}\left(z_{i}\right)\leq \mu_{H}^{+}\left(z_{i}\right),\vartheta_{G}^{-}\left(z_{i}\right)\geq \vartheta_{H}^{-}\left(z_{i}\right),\vartheta_{G}^{+}\left(z_{i}\right)\geq \vartheta_{H}^{+}\left(z_{i}\right)\;\text{for}\;z\;\epsilon \;Z\;\text{.}$.
II. $G^{C}=\left\langle z_{i},\left(\left[\vartheta_{G}^{-}\left(z_{i}\right),\vartheta_{G}^{+}\left(z_{i}\right)\right],\left[\mu_{G}^{-}\left(z_{i}\right),\mu_{G}^{+}\left(z_{i}\right)\right]\right)\right\rangle |z\in Z$.
III. G=H when subjected to G⊆HandH⊆G.

Definition 8 Assume $q_{ij}\;\left(i=1,2,3\ldots ..m\;and\;j=1,2,3\ldots .n\right)$ as a cluster of the IVIHFS based element and $w={\left(w_{1},w_{2},w_{3},\ldots .,w_{n}\right)}^{T}$ be the criteria weight vector by ${CR}_{j}$ (${CR}_{1},{CR}_{2},{CR}_{3\ldots \ldots .n}$). whereas its collective sum measure should not exceed 1. An interval-valued HFS based weighted averaging operator is shown in equation (11).

(11) $$△\left(q_{i}\right)={⨁}_{j=1}^{n}w_{j}q_{ij}$$

If for the evaluated operator weight vectors becomes $w={\left(\frac{1}{w_{1}},\frac{1}{w_{2}},\frac{1}{w_{3}},\ldots .,\frac{1}{w_{n}}\right)}^{T}$ then it reduces to the interval-valued intuitionistic HFS based averaging operator, its aggregated value is calculated by using the formulation mentioned in equation (12).

(12) $$△\left(q_{i}\right)=\left[\left(1-\prod_{i=1}^{n}{\left(1-\mu_{\alpha_{\tau \left(i\right)}}^{-}\right)}^{w_{i}}\right),\left(1-\prod_{i=1}^{n}{\left(1\mu_{\alpha_{\tau \left(i\right)}}^{+}\right)}^{w_{i}}\right),\left(\prod_{i=1}^{n}{\left(\vartheta_{\alpha_{\tau \left(i\right)}}^{-}\right)}^{w_{i}},\prod_{i=1}^{n}{\left(\vartheta_{\alpha_{\tau \left(i\right)}}^{+}\right)}^{w_{i}}\right)|\alpha_{\tau \left(1\right)}\right]$$

Definition 9 Let ‘*F*’ be an IVIHFS belonging to ‘*Z’*, its entropy for ‘F’ is defined as in equation (13).

(13) $$F_{1}\left(B\right)=\frac{1}{n}\sum_{i=1}^{n}\sum_{j=1}^{m}\frac{\min\left\{\mu_{B}^{-}\left(z_{i}^{j}\right),\vartheta_{B}^{-}\left(z_{i}^{j}\right)\right\}+\min\left\{\mu_{B}^{+}\left(z_{i}^{j}\right),\vartheta_{B}^{+}\left(z_{i}^{j}\right)\right\}+\pi_{B}^{-}\left(z_{i}^{j}\right)+\pi_{B}^{+}\left(z_{i}^{j}\right)}{\max\left\{\mu_{B}^{-}\left(z_{i}^{j}\right),\vartheta_{B}^{-}\left(z_{i}^{j}\right)\right\}+\max\left\{\mu_{B}^{+}\left(z_{i}^{j}\right),\vartheta_{B}^{+}\left(z_{i}^{j}\right)\right\}+\pi_{B}^{-}\left(z_{i}^{j}\right)+\pi_{B}^{+}\left(z_{i}^{j}\right)}$$

### 3.3 Data collection

**Fig. 4:**
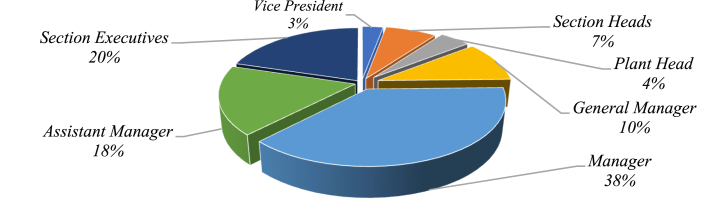
Respondent share.

To validate the feasibility of the developed model, practitioners from food processing units in Northern India are consulted, and brainstorming sessions are held. Experts provided their perspectives on the various actors under consideration and investigated various practicalities associated with them. A questionnaire with rating points is created, and evaluations from field experts are obtained. All of the respondents have more than 5 years of experience working with processed FSCs and related operations. Fig. 4 also includes highlights of the respondents' demography in the form of pie charts to help with visualization.

**Fig. 5:**
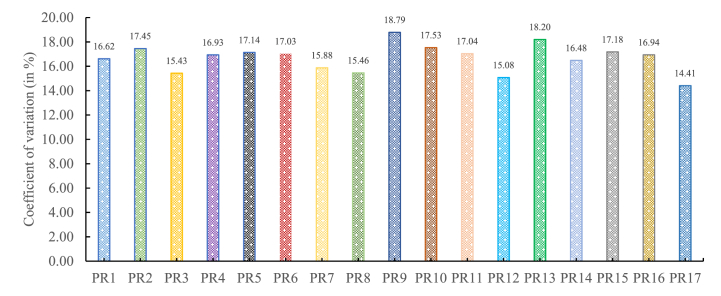
Coefficient of variation in gathered data responses.

The presented work seeds the data inputs gained from the field practitioners on the rating scale. Initially, the gathered data is checked for bias and redundancy by evaluating its coefficient of variation. It is useful for the comparison of the degree of variation in the collected responses (in percentage) (Refer to Fig. 5). Broadly, its range between 10 and 20 percent depicts the zone of acceptability [106]. Furthermore, the gathered is checked for the reliability by evaluating Cronbach alpha coefficient which is 0.825. It infers that gathered data is consistent and reliable enough for analysis. From Fig. 5 it can be inferred that for every enactor of decarbonisation the coefficient of variation of gathered response is within the prescribed range.

### 3.4 Proposed methodology of Entropy Based Interval-Valued Intuitionistic Hesitant Fuzzy Set

The proposed methodology envelopes the entropy measure of the decision critics with the fundamentals of the Interval-Valued Intuitionistic Hesitant Fuzzy Set (IVIHFS). Entropy-based IVIHFS models are robust and reliable enough in domains that are sensitive to decisional inputs. It integrates both the intuitionistic and hesitant fuzzy set theory features distinctly, allowing the decision-makers to express their uncertainty and hesitancy simultaneously. It improves the decision-making process in situations that are uncertain or unpredictable. Furthermore, entropy-based measures quantify the alternatives relative to decisional criteria and felicitate their outranking. Hence, this methodology is well suited for multi-criteria decision-making, where different criteria govern the decision.

**Fig. 6:**
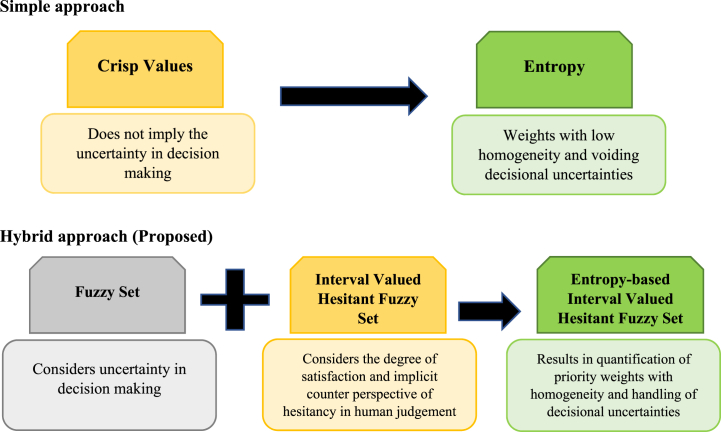
Comparison of simple and proposed hybrid approach.

In comparison to other methodologies like the Analytical Hierarchical Process, where consistency index evaluation limits the decision, Technique of Order Preference by Similarity to Ideal Solution (TOPSIS), an ideal solution determination restricts the solution and methodologies like Interpretative Structural Modelling, Decision Making Trial and Evolution Laboratory cannot be implied to decisional hierarchical models. Furthermore, applying entropy to the evaluation of the relational, hierarchical model provides a complete measure of the distinctness associated with the various decision entities, such as criterion, sub-criterion, and alternatives under consideration. Hence, the ability of the proposed methodology to adapt to a wide range of domains and its flexibility in decision-making enriches its application. Furthermore, Fig. 6 is developed which compares the simple approach and the proposed hybrid approach.

### 3.5 Steps in proposed methodology

This sub-section details the various steps involved in the assessment of developed model by the proposed methodology of Entropy Based Interval-Valued Intuitionistic Hesitant Fuzzy Set.

Step 1 Development of decision matrix

Assuming ‘*AL’* as the set of alternatives ranging as $AL=\{{AL}_{1},{AL}_{2},{AL}_{3}\ldots \ldots ,{AL}_{m}$}and ‘*CR*’ as a set of criteria as $CR=\{{CR}_{1},{CR}_{2},\ldots ,{CR}_{n}$}. Relative to these criteria, weights are depicted as $wg=\left\{{wg}_{1},{wg}_{2},{wg}_{3}\ldots \ldots {wg}_{n}\right\}$, subject to the condition of $0\leq {wg}_{j}\leq 1\;\text{and}\;\sum_{j=1}^{n}{wg}_{j}=1$. Comprising of $Decision\;Makers\;\left(DM\right)=\left\{{dm}_{1},{dm}_{2,}\;{dm}_{3}\ldots ..{dm}_{k}\right\}$, where *‘k’* is total DMs associated with the notions under consideration.

Let the decision matrix provided by the *DMs* be based upon the principles of IVIHFS, where every element is attributed by the set of various IVHFS based numbers showcased as follow (refer to equation (14)):

(14) $$T_{k}={\left[\left({\left(t_{ij}^{k}\right)}^{L},{\left(t_{ij}^{k}\right)}^{U}\right)\right]}_{m*n}\;\text{for}\;\text{all}\;\text{the}\;\text{values}\;\text{of}\;i\;\epsilon \;\left(1,2,3,4\ldots \ldots ,m\right)\;and\;j\;\epsilon \;\left(1,2,3,4\ldots \ldots ,n\right)$$

Step 2 Normalisation of decision matrix

Considering the criterion values (as ${[\left(t_{ij}^{k}\right)}^{L},{\left(t_{ij}^{k}\right)}^{U}]$) in IVHFS-based decision matrices, where ‘*L’* and *‘U’* depicts the lower and upper bounds respectively. $T_{k}={\left({\left(t_{ij}^{k}\right)}^{L},{\left(t_{ij}^{k}\right)}^{U}\right)}_{m*n}$ aimed to measure them as dimensionless units. Decision matrices are normalized to remove, the assessment extremities, whereas $H={\left({\left(h_{ij}^{k}\right)}^{L},{\left(h_{ij}^{k}\right)}^{U}\right)}_{m*n}$ depicts the normalized elements, comprising the formulation as shown in equation (15) [107].

(15) $${\left(h_{ij}^{k}\right)}^{L}=\frac{{\left(t_{ij}^{k}\right)}^{L}}{\sum_{i=1}^{m}{\left(t_{ij}^{k}\right)}^{U}}\;\text{and}\;{\left(h_{ij}^{k}\right)}^{U}=\frac{{\left(t_{ij}^{k}\right)}^{U}}{\sum_{i=1}^{m}{\left(t_{ij}^{k}\right)}^{L}}$$

For the gathered assessments relative to the developed framework, normalized values are obtained are implying equation (15) and showcased in Table 3(a) and 3(b).

**Table 3a:** Normalized decision matrix.

	PR1	PR2	PR3	PR4	PR5	PR6	PR7	PR8	PR9
**H1**	[0.1379,0.4706]; [0.1034,0.4118]; [0.3158,0.2942]; [0.0689,0.3529]; [0.0689,0.1765]	[0.1562,0.4706]; [0.125,0.4118]; [0.0938,0.3529]; [0.0625,0.3529]; [0.0937,0.2941]	[0.2,0.4]; [0.1334,0.35]; [0.1667,0.3]; [0.0667,0.2]; [0.1,0.25]	[0.1562,0.4705]; [0.125,0.4117]; [0.0937,0.3529]; [0.0625,0.3529]; [0.0937,0.2941]	[0.1379,0.4705]; [0.1034,0.4117]; [0.2068,0.2941]; [0.0689,0.3529]; [0.0689,0.1764]	[0.1379,0.4705]; [0.1034,0.4117]; [0.2068,0.2941]; [0.0689,0.3529]; [0.0689,0.1764]	[0.1562,0.4705]; [0.125,0.4117]; [0.0937,0.3529]; [0.0625,0.3529]; [0.0937,0.2941]	[0.2,0.4]; [0.1334,0.35]; [0.1667,0.3]; [0.0667,0.2]; [0.1,0.25]	[0.1379,0.4705]; [0.1034,0.4117]; [0.2068,0.2941]; [0.0689,0.3529]; [0.0689,0.1764]
**H2**	[0.1429,0.4546]; [0.0953,0.3637]; [0.0953,0.2728]; [0.1429,0.3637; [0.0477,0.4546]	[0.1379,0.4705]; [0.1034,0.4117]; [0.2068,0.2941]; [0.0689,0.3529]; [0.0689,0.1764]	[0.1562,0.4705]; [0.125,0.2]; [0.0937,0.15]; [0.0625,0.1]; [0.09375,0.15]	[0.1562,0.4705]; [0.125,0.4117]; [0.0937,0.3529]; [0.0625,0.3529]; [0.0937,0.2941]	[0.1562,0.4705]; [0.125,0.4117]; [0.0937,0.3529]; [0.0625,0.3529]; [0.0937,0.2942]	[0.1379,0.4705]; [0.1034,0.4117]; [0.2068,0.2941]; [0.0689,0.3529]; [0.0689,0.1764]	[0.1428,0.4546]; [0.0952,0.3637]; [0.0952,0.2728]; [0.1428,0.3637]; [0.0476,0.4546]	[0.1562,0.4705]; [0.125,0.4117]; [0.0937,0.3529]; [0.0625,0.3529]; [0.0937,0.2941]	[0.1562,0.4705]; [0.125,0.4118]; [0.0937,0.3529]; [0.0625,0.3529]; [0.0937,0.2942]
**H3**	[0.5,0.4706]; [0.1034,0.4118]; [0.2068,0.2942]; [0.0689,0.3529]; [0.0689,0.1765]	[0.1379,0.1904]; [0.1034,0.1428]; [0.2068,0.2857]; [0.0689,0.0952]; [0.0689,0.0952]	[0.1379,0.4705]; [0.1034,0.1428]; [0.2068,0.2857]; [0.0689,0.0952]; [0.0689,0.0952]	[0.2,0.4]; [0.1334,0.35]; [0.1667,0.3]; [0.0667,0.2]; [0.1,0.25]	[0.2,0.4]; [0.1334,0.35]; [0.1667,0.3]; [0.0667,0.2]; [0.1,0.25]	[0.1562,0.4705]; [0.125,0.4117]; [0.0937,0.3529]; [0.0625,0.3529]; [0.0937,0.2941]	[0.1562,0.4705]; [0.125,0.4117]; [0.0935,0.3529]; [0.0625,0.3529]; [0.0937,0.2941]	[0.1379,0.4705]; [0.1034,0.4117]; [0.2068,0.2941]; [0.0689,0.3529]; [0.0689,0.1764]	[0.1379,0.4705]; [0.1034,0.4117]; [0.2068,0.2941]; [0.0689,0.3529]; [0.0689,0.1764]
**H4**	[0.1429,0.4546]; [0.0953,0.3637]; [0.0953,0.2728]; [0.1429,0.3637]; [0.0477,0.4546]	[0.1379,0.1904]; [0.1034,0.1428]; [0.2068,0.2857]; [0.0689,0.0952]; [0.0689,0.0952]	[0.1379,0.4705]; [0.1034,0.1428]; [0.2068,0.2857]; [0.0689,0.0952]; [0.0689,0.0952]	[0.1562,0.4705]; [0.125,0.4117]; [0.0937,0.3529]; [0.0625,0.3529]; [0.0937,0.2941]	[0.1562,0.4705]; [0.125,0.4117]; [0.0937,0.3529]; [0.0625,0.3529]; [0.0937,0.2941]	[0.2,0.4]; [0.1334,0.35]; [0.1667,0.3]; [0.0667,0.2]; [0.1,0.25]	[0.1428,0.4546]; [0.0952,0.3637]; [0.0952,0.2728]; [0.1428,0.3637]; [0.0476,0.4546]	[0.2,0.4]; [0.1334,0.35]; [0.1667,0.3]; [0.0667,0.2]; [0.1,0.25]	[0.1379,0.4705]; [0.1034,0.4117]; [0.2068,0.2941]; [0.0689,0.3529]; [0.0689,0.1764]
**H5**	[0.1379,0.4705]; [0.1034,0.4117]; [0.2068,0.2941]; [0.0689,0.3529]; [0.0689,0.1764]	[0.15625,0.25]; [0.125,0.2]; [0.09375,0.15]; [0.0625,0.1]; [0.09375,0.15]	[0.1428,0.4545]; [0.0952,0.0952]; [0.0952,0.0952]; [0.1428,0.1428]; [0.0476,0.0476]	[0.1428,0.4546]; [0.0951,0.3637]; [0.0952,0.2728]; [0.1423,0.3637]; [0.0476,0.4546]	[0.1562,0.4705]; [0.125,0.4117]; [0.0937,0.3529]; [0.0625,0.3529]; [0.0937,0.2941]	[0.1562,0.4705]; [0.125,0.4117]; [0.0937,0.3529]; [0.0625,0.3529]; [0.0937,0.2941]	[0.0740,0.3125]; [0.1112,0.3125]; [0.1489,0.375]; [0.1112,0.3125]; [0.1489,0.375]	[0.1562,0.4705]; [0.125,0.4117]; [0.0937,0.3529]; [0.0625,0.3529]; [0.0937,0.2942]	[0.1428,0.4546]; [0.0952,0.3637]; [0.095,0.2728]; [0.1428, 0.3634]; [0.0476,0.4546]
**H6**	[0.1428,0.4545]; [0.0952,0.3636]; [0.0952,0.2727]; [0.1429,0.3637]; [0.0476,0.4546]	[0.1562,0.4705]; [0.125,0.4117]; [0.0937,0.3529]; [0.0625,0.3529]; [0.0937,0.2941]	[0.1379,0.4705]; [0.1034,0.4117]; [0.2068,0.2941]; [0.0689,0.3529]; [0.0689,0.1764]	[0.1379,0.4705]; [0.1034,0.4117]; [0.2068,0.2941]; [0.0689,0.3529]; [0.0689,0.1764]	[0.2,0.4]; [0.1334,0.35]; [0.1667,0.3]; [0.0667,0.2]; [0.1,0.25]	[0.1562,0.4705]; [0.125,0.4117]; [0.0937,0.3529]; [0.0625,0.3529]; [0.0937,0.2941]	[0.1428,0.4545]; [0.0952,0.3637]; [0.0952,0.2728]; [0.1428,0.3637]; [0.0476,0.4546]	[0.1379,0.4705]; [0.1034,0.4117]; [0.2068,0.2941]; [0.0689,0.3529]; [0.0689,0.1764]	[0.1428,0.4546]; [0.0952,0.3634]; [0.0953,0.2728]; [0.1428,0.3634]; [0.0476,0.4545]

**Table 3b:** Normalized decision matrix.

	PR10	PR11	PR12	PR13	PR14	PR15	PR16	PR17
**H1**	[0.2,0.4]; [0.1334,0.35]; [0.1667,0.3]; [0.0667,0.2]; [0.1,0.25]	[0.1428,0.4546]; [0.0952,0.3637]; [0.0952,0.2728]; [0.1428,0.3637]; [0.0476,0.4546]	[0.1562,0.4705]; [0.125,0.4117]; [0.0937,0.3529]; [0.0625,0.3529]; [0.0937,0.2941]	[0.1562,0.4705]; [0.125,0.4118]; [0.0937,0.3529]; [0.0625,0.3529]; [0.0937,0.2941]	[0.15625,0.4705]; [0.125,0.4117]; [0.0937,0.3529]; [0.0625,0.3529]; [0.0937,0.2942]	[0.2,0.4]; [0.1334,0.35]; [0.1667,0.3]; [0.0667,0.2]; [0.1,0.25]	[0.1562,0.4705]; [0.125,0.4117]; [0.0937,0.3529]; [0.0625,0.3529]; [0.0937,0.2941]	[0.2,0.4]; [0.1334,0.35]; [0.1667,0.3]; [0.0667,0.2]; [0.1,0.25]
**H2**	[0.2,0.4]; [0.1334,0.35]; [0.1667,0.3]; [0.0667,0.2]; [0.1,0.25]	[0.1379,0.4705]; [0.1034,0.4117]; [0.2068,0.2941]; [0.0689,0.3529]; [0.0689,0.1764]	[0.1379,0.4705]; [0.1034,0.4117]; [0.2068,0.2941]; [0.0689,0.3529]; [0.0689,0.1764]	[0.2,0.4]; [0.1334,0.35]; [0.1667,0.3]; [0.0667,0.2]; [0.1,0.25]	[0.1562,0.4705]; [0.125,0.4117]; [0.0937,0.3529]; [0.0625,0.3529]; [0.0937,0.29412]	[0.1562,0.4705]; [0.125,0.4118]; [0.09375,0.3529]; [0.0625,0.3529]; [0.0937,0.2942]	[0.1379,0.4705]; [0.1034,0.2413]; [0.2068,0.1724]; [0.0689,0.2068]; [0.0689,0.1034]	[0.1379,0.4705]; [0.1034,0.4117]; [0.2068,0.2941]; [0.0689,0.3529]; [0.0689,0.1764]
**H3**	[0.1562,0.4705]; [0.125,0.4117]; [0.0937,0.3529]; [0.0625,0.3529]; [0.0937,0.2941]	[0.1562,0.4705]; [0.125,0.4117]; [0.0937,0.3529]; [0.0625,0.3529]; [0.0937,0.2941]	[0.1428,0.2307]; [0.0952,0.1538]; [0.0952,0.1538]; [0.1428,0.2307]; [0.0476,0.0769]	[0.1379,0.4705]; [0.1034,0.4117]; [0.2068,0.2941]; [0.0689,0.3529]; [0.0689,0.1764]	[0.2,0.4]; [0.1334,0.35]; [0.1667,0.3]; [0.0667,0.2]; [0.1,0.25]	[0.2,0.4]; [0.1334,0.35]; [0.16667,0.3]; [0.0667,0.2]; [0.1,0.25]	[0.1379,0.4705]; [0.1034,0.4117]; [0.2068,0.2941]; [0.0689,0.3529]; [0.0689,0.1764]	[0.1562,0.4705]; [0.125,0.4117]; [0.0937,0.3529]; [0.0625,0.3529]; [0.0937,0.2941]
**H4**	[0.1379,0.4705]; [0.1034,0.4117]; [0.2068,0.2941]; [0.0689,0.3529]; [0.0689,0.1764]	[0.1428,0.4546]; [0.0952,0.3637]; [0.0952,0.2728]; [0.1428,0.3637]; [0.0476,0.4546]	[0.1428,0.4547]; [0.0952,0.3637]; [0.0952,0.2728]; [0.1428,0.3637]; [0.0476,0.4546]	[0.1379,0.4705]; [0.1034,0.4117]; [0.2068,0.2941]; [0.0689,0.3529]; [0.0689,0.1764]	[0.15625,0.4706]; [0.125,0.4117]; [0.0937,0.3529]; [0.0625,0.3529]; [0.09375,0.2941]	[0.2,0.4]; [0.1334,0.35]; [0.1667,0.3]; [0.0667,0.2]; [0.1,0.25]	[0.1428,0.4546]; [0.0953,0.3637]; [0.0953,0.2728]; [0.1428,0.3637]; [0.0476,0.4546]	[0.1379,0.4705]; [0.1034,0.4117]; [0.2068,0.2941]; [0.0689,0.35294]; [0.0689,0.1764]
**H5**	[0.1379,0.4705]; [0.1034,0.4117]; [0.2068,0.2941]; [0.0689,0.3529]; [0.0689,0.1764]	[0.1379,0.4705]; [0.1034,0.4117]; [0.2068,0.2941]; [0.0689,0.3529]; [0.0689,0.1764]	[0.0740,0.3125]; [0.1112,0.3125]; [0.1482,0.375]; [0.1112,0.3125]; [0.1482,0.375]	[0.2,0.4]; [0.1334,0.35]; [0.1667,0.3]; [0.0667,0.2]; [0.1,0.25]	[0.2,0.4]; [0.1334,0.35]; [0.1667,0.3]; [0.0667,0.2]; [0.1,0.25]	[0.1379,0.4705]; [0.1034,0.4117]; [0.2068,0.2942]; [0.0689,0.3529]; [0.0689,0.1764]	[0.0741,0.3125]; [0.1112,0.3125]; [0.1482,0.375]; [0.1112,0.3125]; [0.1482,0.375]	[0.2,0.4]; [0.1334,0.35]; [0.1667,0.3]; [0.0667,0.2]; [0.1,0.25]
**H6**	[0.1428,0.4546]; [0.0954,0.3637]; [0.0952,0.2728]; [0.1428,0.3637]; [0.0476,0.4546]	[0.1379,0.4705]; [0.1034,0.4117]; [0.2068,0.2941]; [0.0689,0.3529]; [0.0689,0.1764]	[0.1379,0.4705]; [0.1034,0.4117]; [0.2068,0.2941]; [0.0689,0.3529]; [0.0689,0.1764]	[0.1562,0.4705]; [0.125,0.4117]; [0.0937,0.3529]; [0.0625,0.3529]; [0.0937,0.2941]	[0.1379,0.4705]; [0.1034,0.4117]; [0.2068,0.2941]; [0.0689,0.3529]; [0.0689,0.1764]	[0.1562,0.4705]; [0.125,0.4117]; [0.0937,0.3529]; [0.0625,0.3529]; [0.0937,0.2941]	[0.1379,0.4705]; [0.1034,0.4117]; [0.2068,0.2941]; [0.0689,0.3529]; [0.0689,0.1764]	[0.1562,0.4705]; [0.125,0.4117]; [0.0937,0.3529]; [0.0625,0.3529]; [0.0937,0.29411]

In Table 3(a) and 3(b) normalized value of the interval valued hesitant fuzzy sets based linguistics rating is achieved. Inputs during the data collection, have chances that the gathered data may have subsequent extremities. Normalisation is done to remove the data extremities and reduce modalities in the data inputs for further processing.

Step 3 Evaluation of satisfactory and dissatisfactory interval values

Evaluated values of the criterion weights $\left(\left[{\left(h_{1j}^{k}\right)}^{L},{\left(h_{1j}^{k}\right)}^{U}\right]\right)$, $\left(\left[{\left(h_{2j}^{k}\right)}^{L},{\left(h_{2j}^{k}\right)}^{U}\right]\right)$, $\left(\left[{\left(h_{3j}^{k}\right)}^{L},{\left(h_{3j}^{k}\right)}^{U}\right]\right)$ …. $\left({\left(h_{mj}^{k}\right)}^{L},{\left(h_{mj}^{k}\right)}^{U}\right)$ in the normalized decision matrices $H={\left({\left(h_{ij}^{k}\right)}^{L},{\left(h_{ij}^{k}\right)}^{U}\right)}_{m*n}$ infers the evaluation of group assessment. Large interval values $\left[{\left(h_{ij}^{k}\right)}^{L},{\left(h_{ij}^{k}\right)}^{U}\right]$ reveals the scenario of high satisfaction degrees allied with the *‘i*th*’* alternative relative to *‘j*th*’* criteria. Satisfactory and dissatisfactory intervals are depicted by the set of ordered pairs $\left(\left[{\left(\zeta_{ij}^{k}\right)}^{L},{\left(\zeta_{ij}^{k}\right)}^{U}\right]\right)$ and $\left(\left[{\left(\varsigma_{ij}^{k}\right)}^{L},{\left(\varsigma_{ij}^{k}\right)}^{U}\right]\right)$ respectively as shown in equation (16).

(16) $${\left(\zeta_{ij}^{k}\right)}^{L}=\min_{i\;\epsilon \;M}{\left(h_{ij}^{k}\right)}^{U},{\left(\zeta_{ij}^{k}\right)}^{U}=\max_{i\;\epsilon \;M}{\left(h_{ij}^{k}\right)}^{U},{\left(\zeta_{ij}^{k}\right)}^{L}=\min_{i\;\epsilon \;M}$$

It can be rendered that a smaller endpoint ${\left(\zeta_{ij}^{k}\right)}^{L}$/${\left(\zeta_{ij}^{k}\right)}^{U}$ posses the dis-satisfaction degree of the *‘i*th*’* alternative relative to the *‘j*th*’* criterion. In order, to bridge them, transformation allied with the endpoints is formulated in equation (17).

(17) $${\left(\zeta_{ij}^{k}\right)}^{L}=1-\;{\left(\zeta_{ij}^{k}\right)}^{U},{\left(\zeta_{ij}^{k}\right)}^{U}=1-\;{\left(\zeta_{ij}^{k}\right)}^{L}\;\text{for}\;\text{all}\;i\epsilon M,j\epsilon N$$

Step 4 Calculation of aggregated IVIHFS based decision matrix

For the IVIHFS-based group valuation, evaluation of the *‘i*th*’* alternative relative to the *‘j*th*’* criterion underpinning the IVIHFS-based numbers as $\left[{\left(\lambda_{ij}^{k}\right)}^{L},{\left(\lambda_{ij}^{k}\right)}^{U}\;\right],\left[{\left(\eta_{ij}^{k}\right)}^{L},{\left(\eta_{ij}^{k}\right)}^{U}\;\right]$. These numbers are evaluated from the satisfactory and dis-satisfactory intervals $\left(\left[{\left(\zeta_{ij}^{k}\right)}^{L},{\left(\zeta_{ij}^{k}\right)}^{U}\right]\right)$ and $\left(\left[{\left(\varsigma_{ij}^{k}\right)}^{L},{\left(\varsigma_{ij}^{k}\right)}^{U}\right]\right)$, by opting for formulation clustered in a set of equations (18), (19)).

(18) $${\left(\lambda_{ij}^{k}\right)}^{L}=\frac{{\left(\zeta_{ij}^{k}\right)}^{L}}{{\left(\zeta_{ij}^{k}\right)}^{L}+{\left(\zeta_{ij}^{k}\right)}^{U}+{\left(\varsigma_{ij}^{k}\right)}^{L}+{\left(\varsigma_{ij}^{k}\right)}^{U}}\;\text{and}\;{\left(\lambda_{ij}^{k}\right)}^{U}=\frac{{\left(\zeta_{ij}^{k}\right)}^{U}}{{\left(\zeta_{ij}^{k}\right)}^{L}+{\left(\zeta_{ij}^{k}\right)}^{U}+{\left(\varsigma_{ij}^{k}\right)}^{L}+{\left(\varsigma_{ij}^{k}\right)}^{U}}$$

(19) $${\left(\eta_{ij}^{k}\right)}^{L}=\frac{{\left(\varsigma_{ij}^{k}\right)}^{L}}{{\left(\zeta_{ij}^{k}\right)}^{L}+{\left(\zeta_{ij}^{k}\right)}^{U}+{\left(\varsigma_{ij}^{k}\right)}^{L}+{\left(\varsigma_{ij}^{k}\right)}^{U}}\;\text{and}\;{\left(\eta_{ij}^{k}\right)}^{U}=\frac{{\left(\varsigma_{ij}^{k}\right)}^{U}}{{\left(\zeta_{ij}^{k}\right)}^{L}+{\left(\zeta_{ij}^{k}\right)}^{U}+{\left(\varsigma_{ij}^{k}\right)}^{L}+{\left(\varsigma_{ij}^{k}\right)}^{U}}$$

Obtained normalized values of the assessment in the developed normalized decision matrix renders the evaluation of satisfactory and dissatisfactory interval values associated with the ordered pairs of relative alternatives (Refer to equation (16), (17))). Interval, bounds are evaluated relative to the criteria under consideration by engaging a set of equations mentioned 18–19, resulting in values showcased in Table 4.

**Table 4:** Aggregated IVIHFS decision matrix.

	H1	H2	H3	H4	H5	H6
**PR1**	[0.171,0.456]; [0.0668,0.306]; [0.2971,0.4952]; [0.0518,0.1556]	[0.2971,0.4952]; [0.0518,0.1556]; [0.3435,0.4122]; [0.0814,0.1628]	[0.1451,0.3869]; [0.0567,0.4111]; [0.3435,0.4122]; [0.0814,0.1628]	[0.2971,0.4952]; [0.0518,0.1556]; [0.3435,0.4122]; [0.0814,0.1628]	[0.1912,0.5098]; [0.0747,0.2241]; [0.1912,0.5098]; [0.0747,0.2241]	[0.2971,0.4952]; [0.0518,0.1556]; [0.3435,0.4122]; [0.0814,0.1628]
**PR2**	[0.299,0.4785]; [0.0635,0.1588]; [0.299,0.4785]; [0.0635,0.1588]	[0.1912,0.5098]; [0.0747,0.2241]; [0.2971,0.4952]; [0.0518,0.1556]	[0.145,0.435]; [0.105,0.315]; [0.1544,0.4632]; [0.0955,0.2867]	[0.145,0.435]; [0.105,0.315]; [0.1909,0.5727]; [0.059,0.1772]	[0.1758,0.4395]; [0.1098,0.2747]; [0.1544,0.4632]; [0.0955,0.2867]	[0.2308,0.3694]; [0.277,0.1226]; [0.2386,0.3977]; [0.2386,0.125]
**PR3**	[0.2307,0.4615]; [0.0769,0.2307]; [0.2307,0.4615]; [0.0769,0.2307]	[0.1266,0.5961]; [0.0791,0.1979]; [0.1131,0.5591]; [0.0819,0.2458]	[0.1131,0.5591]; [0.0819,0.2458]; [0.1131,0.5591]; [0.0819,0.2458]	[0.1131,0.5591]; [0.0819,0.2458]; [0.1131,0.5591]; [0.0819,0.2458]	[0.0687,0.6562]; [0.0687,0.2062]; [0.0845,0.5771]; [0.0845,0.2537]	[0.1912,0.5098]; [0.0747,0.2241]; [0.1912,0.5098]; [0.0747,0.2241]
**PR4**	[0.299,0.4785]; [0.0635,0.1588]; [0.2307,0.4615]; [0.0769,0.2307]	[0.299,0.4785]; [0.0635,0.1588]; [0.299,0.4785]; [0.0635,0.1588]	[0.2307,0.4615]; [0.0769,0.2307]; [0.299,0.4785]; [0.0635,0.1588]	[0.299,0.4785]; [0.0635,0.1588]; [0.2307,0.4615]; [0.0769,0.2307]	[0.2971,0.4952]; [0.0518,0.1556]; [0.299,0.4785]; [0.0635,0.1588]	[0.1912,0.5098]; [0.0747,0.2241]; [0.2971,0.4952]; [0.0518,0.1556]
**PR5**	[0.1912,0.5098]; [0.0747,0.2241]; [0.2971,0.4952]; [0.0518,0.1556]	[0.299,0.4785]; [0.0635,0.1588]; [0.2307,0.4615]; [0.0769,0.2307]	[0.2307,0.4615]; [0.0769,0.2307]; [0.2307,0.4615]; [0.0769,0.2307]	[0.299,0.4785]; [0.0635,0.1588]; [0.299,0.4785]; [0.0635,0.1588]	[0.299,0.4785]; [0.0635,0.1588]; [0.2307,0.4615]; [0.0769,0.2307]	[0.2307,0.4615]; [0.0769,0.2307]; [0.299,0.4785]; [0.0635,0.1588]
**PR6**	[0.1912,0.5098]; [0.0747,0.2241]; [0.3435,0.4122]; [0.0814,0.1628]	[0.1912,0.5098]; [0.0747,0.2241]; [0.1912,0.5098]; [0.0747,0.2241]	[0.299,0.4785]; [0.0635,0.1588]; [0.299,0.4785]; [0.0635,0.1588]	[0.2307,0.4615]; [0.0769,0.2307]; [0.299,0.4785]; [0.0635,0.1588]	[0.299,0.4785]; [0.0635,0.1588]; [0.299,0.4785]; [0.0635,0.1588]	[0.299,0.4785]; [0.0635,0.1588]; [0.299,0.4785]; [0.0635,0.1588]
**PR7**	[0.299,0.4785]; [0.0635,0.1588]; [0.299,0.4785]; [0.0635,0.1588]	[0.2971,0.4952]; [0.0518,0.1556]; [0.3435,0.4122]; [0.0814,0.1628]	[0.299,0.4785]; [0.0635,0.1588]; [0.2307,0.4615]; [0.0769,0.2307]	[0.2971,0.4952]; [0.0518,0.1556]; [0.3435,0.4122]; [0.0814,0.1628]	[0.3435,0.4122]; [0.0814,0.1628]; [0.1912,0.5098]; [0.0747,0.2241]	[0.2971,0.4952]; [0.0518,0.1556]; [0.3435,0.4122]; [0.0814,0.1628]
**PR8**	[0.2307,0.4615]; [0.0769,0.2307]; [0.299,0.4785]; [0.0635,0.1588]	[0.299,0.4785]; [0.0635,0.1588]; [0.2307,0.4615]; [0.0769,0.2307]	[0.1912,0.5098]; [0.0747,0.2241]; [0.2971,0.4952]; [0.0518,0.1556]	[0.2,0.4]; [0.2,0.2]; [0.2692,0.4487]; [0.141,0.141]	[0.299,0.4785]; [0.0635,0.1588]; [0.299,0.4785]; [0.0635,0.1588]	[0.1912,0.5098]; [0.0747,0.2241]; [0.1912,0.5098]; [0.0747,0.2241]
**PR9**	[0.1912,0.5098]; [0.0747,0.2241]; [0.1912,0.5098]; [0.0747,0.2241]	[0.299,0.4785]; [0.0635,0.1588]; [0.299,0.4785]; [0.0635,0.1588]	[0.1912,0.5098]; [0.0747,0.2241]; [0.1912,0.5098]; [0.0747,0.2241]	[0.1912,0.5098]; [0.0747,0.2241]; [0.299,0.4785]; [0.0635,0.1588]	[0.2971,0.4952]; [0.0518,0.1556]; [0.2971,0.4952]; [0.0518,0.1556]	[0.2971,0.4952]; [0.0518,0.1556]; [0.299,0.4785]; [0.0635,0.1588]
**PR10**	[0.2743,0.4573]; [0.067,0.2012]; [0.2882,0.4804]; [0.066,0.1651]	[0.2307,0.4615]; [0.0769,0.2307]; [0.2307,0.4615]; [0.0769,0.2307]	[0.299,0.4785]; [0.0635,0.1588]; [0.2307,0.4615]; [0.0769,0.2307]	[0.1912,0.5098]; [0.0747,0.2241]; [0.1912,0.5098]; [0.0747,0.2241]	[0.1912,0.5098]; [0.0747,0.2241]; [0.299,0.4785]; [0.0635,0.1588]	[0.2971,0.4952]; [0.0518,0.1556]; [0.2307,0.4615]; [0.0769,0.2307]
**PR11**	[0.2971,0.4952]; [0.0518,0.1556]; [0.2971,0.4952]; [0.0518,0.1556]	[0.1912,0.5098]; [0.0747,0.2241]; [0.2971,0.4952]; [0.0518,0.1556]	[0.299,0.4785]; [0.0635,0.1588]; [0.299,0.4785]; [0.0635,0.1588]	[0.2971,0.4952]; [0.0518,0.1556]; [0.2971,0.4952]; [0.0518,0.1556]	[0.1912,0.5098]; [0.0747,0.2241]; [0.2971,0.4952]; [0.0518,0.1556]	[0.1912,0.5098]; [0.0747,0.2241]; [0.2971,0.4952]; [0.0518,0.1556]
**PR12**	[0.299,0.4785]; [0.0635,0.1588]; [0.2307,0.4615]; [0.0769,0.2307]	[0.1912,0.5098]; [0.0747,0.2241]; [0.2971,0.4952]; [0.0518,0.1556]	[0.1544,0.4632]; [0.0955,0.2867]; [0.145,0.435]; [0.105,0.315]	[0.2971,0.4952]; [0.0518,0.1556]; [0.3435,0.4122]; [0.0814,0.1628]	[0.3435,0.4122]; [0.0814,0.1628]; [0.3435,0.4122]; [0.0814,0.1628]	[0.1912,0.5098]; [0.0747,0.2241]; [0.1912,0.5098]; [0.0747,0.2241]
**PR13**	[0.299,0.4785]; [0.0635,0.1588]; [0.299,0.4785]; [0.0635,0.1588]	[0.2307,0.4615]; [0.0769,0.2307]; [0.2307,0.4615]; [0.0769,0.2307]	[0.1912,0.5098]; [0.0747,0.2241]; [0.299,0.4785]; [0.0635,0.1588]	[0.1912,0.5098]; [0.0747,0.2241]; [0.1912,0.5098]; [0.0747,0.2241]	[0.2307,0.4615]; [0.0769,0.2307]; [0.299,0.4785]; [0.0635,0.1588]	[0.299,0.4785]; [0.0635,0.1588]; [0.299,0.4785]; [0.0635,0.1588]
**PR14**	[0.299,0.4785]; [0.0635,0.1588]; [0.1912,0.5098]; [0.0747,0.2241]	[0.299,0.4785]; [0.0635,0.1588]; [0.299,0.4785]; [0.0635,0.1588]	[0.2307,0.4615]; [0.0769,0.2307]; [0.2307,0.4615]; [0.0769,0.2307]	[0.299,0.4785]; [0.0635,0.1588]; [0.299,0.4785]; [0.0635,0.1588]	[0.2307,0.4615]; [0.0769,0.2307]; [0.299,0.4785]; [0.0635,0.1588]	[0.1912,0.5098]; [0.0747,0.2241]; [0.299,0.4785]; [0.0635,0.1588]
**PR15**	[0.2307,0.4615]; [0.0769,0.2307]; [0.2307,0.4615]; [0.0769,0.2307]	[0.299,0.4785]; [0.0635,0.1588]; [0.1912,0.5098]; [0.0747,0.2241]	[0.2307,0.4615]; [0.0769,0.2307]; [0.2307,0.4615]; [0.0769,0.2307]	[0.2283,0.4566]; [0.0787,0.2362]; [0.2432,0.4864]; [0.09,0.1801]	[0.1912,0.5098]; [0.0747,0.2241]; [0.3435,0.4122]; [0.0814,0.1628]	[0.299,0.4785]; [0.0635,0.1588]; [0.2307,0.4615]; [0.0769,0.2307]
**PR16**	[0.299,0.4785]; [0.0635,0.1588]; [0.299,0.4785]; [0.0635,0.1588]	[0.1217,0.5536]; [0.0811,0.2434]; [0.1847,0.5565]; [0.0739,0.1847]	[0.1912,0.5098]; [0.0747,0.2241]; [0.2971,0.4952]; [0.0518,0.1556]	[0.2971,0.4952]; [0.0518,0.1556]; [0.1912,0.5098]; [0.0747,0.2241]	[0.3435,0.4122]; [0.0814,0.1628]; [0.2971,0.4952]; [0.0518,0.1556]	[0.1912,0.5098]; [0.0747,0.2241]; [0.1912,0.5098]; [0.0747,0.2241]
**PR17**	[0.2307,0.4615]; [0.0769,0.2307]; [0.2307,0.4615]; [0.0769,0.2307]	[0.1912,0.5098]; [0.0747,0.2241]; [0.2307,0.4615]; [0.0769,0.2307]	[0.299,0.4785]; [0.0635,0.1588]; [0.1912,0.5098]; [0.0747,0.2241]	[0.1912,0.5098]; [0.0747,0.2241]; [0.299,0.4785]; [0.0635,0.1588]	[0.2307,0.4615]; [0.0769,0.2307]; [0.2826,0.4522]; [0.0662,0.1988]	[0.299,0.4785]; [0.0635,0.1588]; [0.1912,0.5098]; [0.0747,0.2241]

In Table 4 based upon equation (17), (18), (19)), aggregated IVIHFS decision matrix is evaluated. This matrix aggregates the data inputs relative to every criterion and alternative respectively. Specifically, for every criterion considered in the developed model depicted as *H1, H2, H3, H4, H5* and *H6* an aggregated value is evaluated relative to every enactors *PR1, PR2, …..,PR17*.

Step 5 Evaluation of entropy values

The evaluated values grounds the development of group decision matrix obeying the fundamentals of IVIHFS and structured in the matrix depicted by notation *‘L’* (refer equation (20))

(20) $$L=\left[\begin{array}{cccc} AL & {CR}_{1} & & {CR}_{n}\\ {AL}_{1} & \left[{\left(\lambda_{11}^{k}\right)}^{L},{\left(\lambda_{11}^{k}\right)}^{U}\;\right],\left[{\left(\eta_{11}^{k}\right)}^{L},{\left(\eta_{11}^{k}\right)}^{U}\;\right] & \cdots & \left[{\left(\lambda_{1n}^{k}\right)}^{L},{\left(\lambda_{1n}^{k}\right)}^{U}\;\right],\left[{\left(\eta_{1n}^{k}\right)}^{L},{\left(\eta_{1n}^{k}\right)}^{U}\;\right]\\ & \vdots & \ddots & \vdots \\ {AL}_{m} & \left[{\left(\lambda_{m1}^{k}\right)}^{L},{\left(\lambda_{m1}^{k}\right)}^{U}\;\right],\left[{\left(\eta_{m1}^{k}\right)}^{L},{\left(\eta_{m1}^{k}\right)}^{U}\;\right] & \cdots & \left[{\left(\lambda_{mn}^{k}\right)}^{L},{\left(\lambda_{mn}^{k}\right)}^{U}\;\right],\left[{\left(\eta_{mn}^{k}\right)}^{L},{\left(\eta_{mn}^{k}\right)}^{U}\;\right] \end{array}\right]$$

Relative to these criteria, weights are depicted as $wg={\left\{{wg}_{1},{wg}_{2},{wg}_{3}\ldots \ldots {wg}_{n}\right\}}^{T}$, subject to the condition of $0\leq {wg}_{j}\leq 1\;\text{and}\;\sum_{j=1}^{n}{wg}_{j}=1$. IVIHF based entropy measure is determinant of optimal weight (Refer equation (21)).

(21) $${wg}_{j}=\frac{1}{\left(n-W\right)}*\left(1-s_{j}\right),\text{where},j=1,2,3,\ldots \ldots .,n$$

$s_{j}=\sum_{i=1}^{m}h_{ij}\;\text{and}\;W=\sum_{i=1}^{m}s_{j}$ yielding the normalized value associated with entropy $\left(h_{ij}\right)=\frac{{ER}_{ij}}{\max\;{\left(ER\right)}_{ij}}$. Aggregated values of IVIHFS elements, seeds in the calculation of entropy values for the relative value of alternatives, and criterion, implying formulation rendered in equation (16), resulting in values mentioned in Table 5.

**Table 5:** Evaluated entropy values.

	H1	H2	H3	H4	H5	H6
**PR1**	[0.1406,0.1122]	[0.1122,0.1181]	[0.1546,0.1181]	[0.1122,0.1181]	[0.1272,0.1272]	[0.1122,0.1181]
**PR2**	[0.1169,0.1169]	[0.1908,0.1145]	[0.1527,0.1452]	[0.15,0.117]	[0.1431,0.1427]	[0.1372,0.1391]
**PR3**	[0.1313,0.1313]	[0.1853,0.1349]	[0.1349,0.1349]	[0.1325,0.1325]	[0.1234,0.1345]	[0.1275,0.1275]
**PR4**	[0.1169,0.1313]	[0.1719,0.1169]	[0.1313,0.1169]	[0.1148,0.129]	[0.1125,0.1148]	[0.1275,0.1125]
**PR5**	[0.1298,0.1145]	[0.1719,0.1313]	[0.1313,0.1313]	[0.1148,0.1148]	[0.1148,0.129]	[0.129,0.1148]
**PR6**	[0.1298,0.1204]	[0.1908,0.1298]	[0.1169,0.1169]	[0.129,0.1148]	[0.1148,0.1148]	[0.1148,0.1148]
**PR7**	[0.1169,0.1169]	[0.1684,0.1204]	[0.1169,0.1313]	[0.1125,0.1183]	[0.1183,0.1275]	[0.1125,0.1183]
**PR8**	[0.1313,0.1169]	[0.1719,0.1313]	[0.1298,0.1145]	[0.1461,0.1246]	[0.1148,0.1148]	[0.1275,0.1275]
**PR9**	[0.1298,0.1298]	[0.1719,0.1169]	[0.1298,0.1298]	[0.1275,0.1148]	[0.1125,0.1125]	[0.1125,0.1148]
**PR10**	[0.1245,0.1183]	[0.1931,0.1313]	[0.1169,0.1313]	[0.1275,0.1275]	[0.1275,0.1148]	[0.1125,0.129]
**PR11**	[0.1145,0.1145]	[0.1908,0.1145]	[0.1169,0.1169]	[0.1125,0.1125]	[0.1275,0.1125]	[0.1275,0.1125]
**PR12**	[0.1169,0.1313]	[0.1908,0.1145]	[0.1452,0.1527]	[0.1125,0.1183]	[0.1183,0.1183]	[0.1275,0.1275]
**PR13**	[0.1169,0.1169]	[0.1931,0.1313]	[0.1298,0.1169]	[0.1275,0.1275]	[0.129,0.1148]	[0.1148,0.1148]
**PR14**	[0.1169,0.1298]	[0.1719,0.1169]	[0.1313,0.1313]	[0.1148,0.1148]	[0.129,0.1148]	[0.1275,0.1148]
**PR15**	[0.1313,0.1313]	[0.1719,0.1298]	[0.1313,0.1313]	[0.1303,0.1226]	[0.1275,0.1183]	[0.1148,0.129]
**PR16**	[0.1169,0.1169]	[0.1976,0.1228]	[0.1298,0.1145]	[0.1125,0.1275]	[0.1183,0.1125]	[0.1275,0.1275]
**PR17**	[0.1313,0.1313]	[0.1908,0.1313]	[0.1169,0.1298]	[0.1275,0.1148]	[0.129,0.1217]	[0.1148,0.1275]

In Table 5, aggregated values of IVIHFS are used to evaluate the entropy values. These values are again evaluated for every criterion (*H1, H2, H3 …..,H6*) relative to every enactor under consideration (*PR1, PR2, ….,PR16,PR17*). For the same equation (20), (21)) are used and evaluated values are detailed in Table 4.

Step 6 Assessment of preference scores for enactors

By implying the aggregating operator for determination of $△\left(q_{i}\right)$ of the alternatives, IVIHF based preferences values are evaluated as shown in equation (22).

(22) $$△\left(q_{i}\right)=Q=\left[\left(\lambda_{k}^{-}-\;\eta_{k}^{-}\right)+\left(\lambda_{k}^{+}-\;\eta_{k}^{+}\right)\right]\;\text{and}\;S\left(△\left(q_{i}\right)\right)=\frac{\sum_{k=1}^{\#Q}\left[\left(\lambda_{k}^{-}-\;\eta_{k}^{-}\right)+\left(\lambda_{k}^{+}-\;\eta_{k}^{+}\right)\right]}{\#Q}$$

where ‘*#Q*’ depicts the cluster of elements in ‘*Q*. Based upon the above valuations, alternatives are ranked in the descending order of obtained score values [$S\left(△\left(q_{i}\right)\right)$].

Based on equation (22), overall preference score is evaluated of for every enactor under consideration distinctly. Evaluated values are clustered in Table 6 along with the ranking of every enactor.

**Table 6:** Overall preference score of enactors.

Aggregated values		Overall score of preference			Rank
$△\left(q_{1}\right)$	[(0.2281,0.466); (0.058,0.2401); (0.3049,0.4382); (0.0734; 0.1691)]	***PR1***	$sum△\left(q_{1}\right)$	0.2241	7
$△\left(q_{2}\right)$	[(0.1963,0.4355); (0.1254,0.2341); (0.2168,0.4724); (0.1034; 0.1989)]	***PR2***	$sum△\left(q_{2}\right)$	0.1648	17
$△\left(q_{3}\right)$	[(0.1402,0.5512); (0.0754,0.222); (0.1411,0.5299); (0.0785; 0.236)]	***PR3***	$sum△\left(q_{3}\right)$	0.1877	16
$△\left(q_{4}\right)$	[(0.2639,0.4764); (0.0643,0.1791); (0.2702,0.4679); (0.0647; 0.1803)]	***PR4***	$sum△\left(q_{4}\right)$	0.2475	4
$△\left(q_{5}\right)$	[(0.2524,0.4706); (0.0686,0.1921); (0.2618,0.4658); (0.0664; 0.1893)]	***PR5***	$sum△\left(q_{5}\right)$	0.2335	10
$△\left(q_{6}\right)$	[(0.2511,0.4775); (0.0677,0.1878); (0.2888,0.4638); (0.0666; 0.1646)]	***PR6***	$sum△\left(q_{6}\right)$	0.2486	3
$△\left(q_{7}\right)$	[(0.3004,0.4678); (0.0598,0.1547); (0.2863,0.4434); (0.0746; 0.1815)]	***PR7***	$sum△\left(q_{7}\right)$	0.2568	2
$△\left(q_{8}\right)$	[(0.229,0.4664); (0.0927,0.1978); (0.2619,0.4722); (0.0773; 0.1728)]	***PR8***	$sum△\left(q_{8}\right)$	0.2222	12
$△\left(q_{9}\right)$	[(0.239,0.4929); (0.0639,0.1886); (0.2576,0.4847); (0.064; 0.178)]	***PR9***	$sum△\left(q_{9}\right)$	0.2450	5
$△\left(q_{10}\right)$	[(0.2446,0.4791); (0.0662,0.1941); (0.2421,0.4687); (0.0707; 0.2019)]	***PR10***	$sum△\left(q_{10}\right)$	0.2254	11
$△\left(q_{11}\right)$	[(0.2438,0.4915); (0.0634,0.1857); (0.2918,0.4845); (0.0527; 0.1529)]	***PR11***	$sum△\left(q_{11}\right)$	0.2642	1
$△\left(q_{12}\right)$	[(0.2472,0.4698); (0.072,0.1983); (0.2555,0.4462); (0.0781; 0.2088)]	***PR12***	$sum△\left(q_{12}\right)$	0.2154	14
$△\left(q_{13}\right)$	[(0.2376,0.4768); (0.0699,0.1997); (0.2676,0.4741); (0.0657; 0.1761)]	***PR13***	$sum△\left(q_{13}\right)$	0.2362	9
$△\left(q_{14}\right)$	[(0.2524,0.4705); (0.0686,0.1921); (0.2642,0.4735); (0.0663; 0.1797)]	***PR14***	$sum△\left(q_{14}\right)$	0.2385	8
$△\left(q_{15}\right)$	[(0.2401,0.4668); (0.0712,0.2052); (0.2443,0.4564); (0.0779; 0.2055)]	***PR15***	$sum△\left(q_{15}\right)$	0.2120	15
$△\left(q_{16}\right)$	[(0.2452,0.4839); (0.0692,0.1893); (0.2433,0.4977); (0.0633; 0.1805)]	***PR16***	$sum△\left(q_{16}\right)$	0.2420	6
$△\left(q_{17}\right)$	[(0.2392,0.4745); (0.07,0.2002); (0.2343,0.4724); (0.0704; 0.2065)]	***PR17***	$sum△\left(q_{17}\right)$	0.2183	13

## 4 Results and discussion

The analysis of the developed framework of decarbonisation enactors in the processed food supply chain is analysed. For the same, the methodology of entropy-based measures is integrated with the fundamentals of IVIHFS, resulting in the prioritisation of enactors. The overall preference score for each enactor that was obtained is detailed in Table 6.

**Fig. 7:**
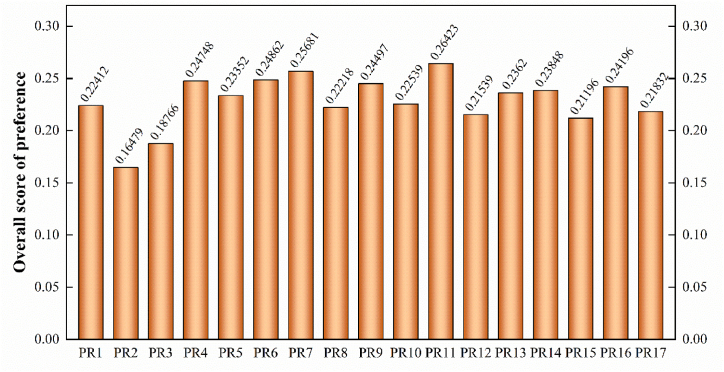
Plot of the evaluated values of decarbonisation enactors.

It can be inferred from the values in Table 5 that the overall preference score outranks the enactors. Based upon it, ‘*Traceability and monitoring of the food items (PR11)*’ outranks highly and secures the highest primacy. It is followed by the enactor ‘*Operations favouring economic viability (PR7)’* and ‘*Minimised wastage during production stages (PR6)*’. It is least significant for the enactor ‘*Incitation of ISO 20400 protocols (PR2)*’. Furthermore, for a better analysis of the outcomes, a graphical plot of the overall preference score is made in Fig. 7.

Outcomes reveal that the enactor ‘*traceability and monitoring of the food items (PR11*)' poses a high significance for switching towards sustainable avenues in FSCs. Enabling Traceability in FSC dynamics improves the monitoring and visibility of its functional entities. It accumulates and overviews the carbon footprint being generated at the various operational tiers of FSCs. Hence, a traceability-enabled FSC is reliant enough to handle multiple remote operations and ensure its global expansion. It validates various sustainability claims relative to product, process, and operating mechanisms. The need arises to develop strong product monitoring and feedback mechanisms, have more sustainable product demands, and make consumers aware of decarbonisation perspectives. Nowadays, in business-to-business and business-to-consumer industries, investors and regulators are demanding sustainable production and consumption. Hence, a traceability mechanism to gather consumer confidence and mark product transition from traditional to sustainable means must be evolved. Some traceability-based digitalisation prospects in FSC need to be assessed to strengthen social, economic and environmental perspectives.

Furthermore, in FSC, it is essential to have ‘*operations favouring economic viability (PR7)*'. This is necessary to ensure food safety and security in the FSC dynamics. It is also ranked primarily among the enactors under consideration by the proposed methodology. Operations that do not favour economic viability often cause widespread product wastage, high product costs, and customer resentment in this competitive market. Hence, to promote social equitability and ensure food to all, operations favouring economic viability must be underpinned in FSC. Even though economic operations result in the optimised selection of various parameters governing the journey from farm to fork, curtailing the carbon footprint generated in the FSC. It is an enabler of ‘*minimised wastage during production stages (PR6)',* which improves product quality and reduces its costs. In terms of sustainability, it is a determinant of economic feasibility, social upbringing, and environmental viability. Curtailing wastage minimises the volume of landfills, preserves resources, and promotes decarbonisation in operations.

Processed FSC begins with customer demand, which is equivalent to the procurement. An inference can be grounded from proposed methodology outcomes that ‘*Circular procurement practices (PR4)*' play an important role. It focuses on making procurement procedures environment-friendly and reducing the wastage generated. These practices leverage the decarbonisation perspective at the first operational tiers of FSCs. In processed FSCs, procurement of raw-material binding with sustainable goals provides social, economic, and environmental benefits, summing up in profitability. It adds value to FSC operations. It is an initial approach to step closer towards the SDG-12 promoting sustainable production-consumption.

Furthermore, an ‘*innovative solution to promote sustainable practices' (PR9)* is required as FSC comprises an interconnected network of operations. Consistent developments aim to extend the product's shelf life, minimise transportation losses, reduce costs, and lay the foundation for digitalisation and decarbonisation. Innovations are necessary to establish the operational principles associated with sustainable FSC processing procedures.

## 5 Sensitivity analysis

**Fig. 8:**
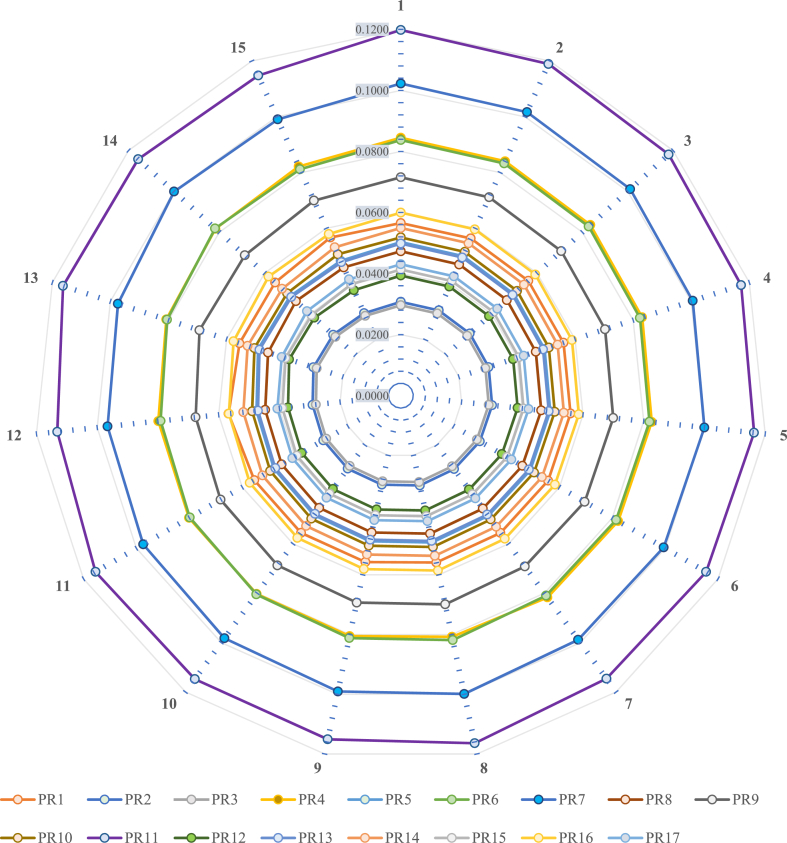
Outcomes of the sensitivity analysis.

As the inputs made to the proposed methodology are based upon the response gained from the field practitioners. Hence, the need arises to check the robustness and feasibility of the obtained priority of enactors from Entropy based – IVIHFS by carrying out sensitivity analysis [108]. For the same, fifteen experiments are performed consecutively, to investigate the influence of priority weights on the value of enactors under consideration. Obtained values are plotted in Fig. 7 relative to every experimental trial. In Fig. 8 relative to every enactor under consideration its preference score value is given some weightage and its repeated consecutively for set of 15 experiments. Similarly, it is done for the all enactors for the same set of experiments. It is done to check the robustness of results under varying condition to aid reliable decision making process.

It is remarkable from Fig. 8 that *‘PR11’* secures highest priority among the all other enactors of sustainability. By assigning priority weights in every experiment, variation is observed and data plots are made. It can be visualised for all 17 enactors from *PR1, PR2, ….., PR17* data plots are made and it showcases minimal variation when the experiments are made consecutively. Hence, an inference can be grounded that obtained values from the proposed methodology are robust and reliable enough to ground decision-making.

## 6 Conclusions

The presented work is aimed at decarbonising the existing dynamics of the processed food supply chain and making it ready for responsible production-consumption mechanisms. For the same, its different enactors are identified and mapped with the event of its occurrence in the food supply chain. It yields the development of a relational, hierarchical model, which is analysed with the methodology of entropy-based measure of interval-valued hesitant fuzzy sets. It results in the quantification of the enactors in terms of their degree of preference for the decarbonisation of existing mechanisms. Furthermore, results are validated for their robustness by carrying out the sensitivity analysis.

It can be concluded from the results that the enactor ‘Traceability and monitoring of the food items (PR11)’ secures the highest priority in sequence to decarbonise the FSCs. It reveals that traceability-enabled FSC prompts the improved monitoring of the production-consumption mechanism, validates sustainability claims, and aids in the reduction of the carbon footprint being generated. Transparency in operations brings up the area where decarbonisation efforts need to be ramped up. Traceability measures the felicities of the transition from traditional to decarbonised food supply chains. Its further extension with the field advancements of digital technologies broadens its scope to enable remote monitoring of the carbon footprint being generated at various stages of the food supply chain. This aids the decision-making process to decarbonise the food supply chain dynamics and make production consumption mechanisms more sustainable.

Furthermore, ‘operations favouring economic viability (PR7)’ also pose as an important enactor to ensure food safety security and reduce wastage at various stages of the food supply chain. It is not restricted to economic viability, but it is an enabler of sustainability and an efficient food supply chain. It aids the streamlining of various operations, reduces wastage, and optimises the utility of resources, leading to a reduced carbon footprint being generated. Endorsing the ‘Minimised wastage during production stages (PR6)’ is essential to decarbonise the food supply chain practices.

The initial stages of the food supply chain begin with the inception of customer demand and procurement, which highlights the importance of enactor ‘Circular procurement practices (PR4)’. It establishes an earlier platform to make production consumption more sustainable in food supply chains. Furthermore, complying with the circularity in procurement practices leads the way towards the decarbonisation of food supply chain operations. Enacting the ‘Innovative solution to promote sustainable practices (PR9)’ marks the way towards the design and development of operations that comply with the decarbonisation portfolios. Consistent innovations, in terms of improving product shelf life, digitalisation of operations, and minimisation of losses, significantly curtail the carbon footprint being generated. It felicities the customisation of decarbonisation efforts relative to the food supply chain under consideration. In a nutshell, the presented work carves the way towards decarbonisation and making food supply chains more responsible towards production consumption. It marks the transition of the traditional food supply chain to a decarbonised and future-ready food supply chain by mediating through the framework of enactors under consideration. The secured priority of the enactors acts as a roadmap towards the journey to responsible production-consumption mechanisms.

## 7 Work implications

The presented work finds its implication in the multi-disciplinary domain. It has the potential to extend its implications to industrial, academic, and governmental portfolios. This study comprehensively overlays the concept of decarbonisation and its importance in the food supply chain operations. It overviews its key potential and relativity with the industry, academia and concerned government bodies. Specifically, in the context of developing economies, it is a common notion that decarbonisation procedures are always difficult to take off and require huge working capital. Owing to the same, this work deliberates the various enactors which are specified to the operational tiers of food supply chains, marking a roadmap towards decarbonisation. Some more implications of the presented work are explained as follows:

• The derived priority of the enactors of decarbonisation provides a strong foundation that felicitates policy development in the domain of decarbonisation of food supply chains. It advocates the various decisions within the food supply chain to identify the specific areas where decarbonisation interventions are required. The developed framework empowers managers to prioritise decarbonisation actions at different stages of the food supply chains.
• The identified enactors of decarbonisation act as a roadmap for the industrial arena to transform their existing practices towards responsible production-consumption mechanisms. It facilitates a gradual transition towards sustainable development goals by deliberately reducing the carbon footprint being generated.
• Decision-makers can use the outcomes of the presented work to make informed choices and overcome the risks and issues hindering the transition towards decarbonised food supply chains. Government bodies can ramp up the necessary support system and address mechanisms to overcome the issues in this transformation.
• Various governmental bodies and concerned organisations can collaborate to develop product traceability mechanisms in food supply chains. This mechanism should have the potential to unite the multiple stakeholders of food supply chains and monitor the product progress and carbon footprint being generated. It can facilitate the decision-making process at strategic, tactical and operational levels, along with its adjustment with time.
• Academicians can use the outranked priorities of enactors to develop various measures to assess the decarbonisation performance of food supply chain performance systems. It can be based upon the outranked preferences of the enactors under consideration, which can reveal the efficiency of the roadmap towards the decarbonisation and fulfilment of sustainability objectives.

## 8 Future scope

The presented work can be extended in future to safeguard the decarbonisation interests in food supply chain and achievement of sustainability goals. Various different regional studies can be to explore the potential of decarbonisation strategies. It can yield the exchange of various effective measures which can promote responsible production-consumption mechanisms. Furthermore, feasibility of the integration of the different technologies like Internet of Things (IoT), blockchain, artificial intelligence with the supply chain can be explored. It can enhance the transparency and effectiveness of the decarbonisation practices. Some simulation or predictive analysis-based tools can be used to assess the long-term impact of the decarbonisation strategies. In continuation to same, product lifecycle assessment can be made to determine its end of life and carbon footprint being generated during its transition at various stages of supply chain. In order to make the change economically viable, its analysis can be conducted to safeguard the economic interests of stakeholders.

## CRediT authorship contribution statement

**Janpriy Sharma:** Writing – original draft. **Shweta Singh:** Writing – review & editing, Investigation, Formal analysis, Conceptualization. **Mohit Tyagi:** Visualization, Validation, Supervision. **Satvasheel Powar:** Writing – review & editing, Visualization, Validation, Supervision.

## Declaration of competing interest

The authors declare that they have no known competing financial interests or personal relationships that could have appeared to influence the work reported in this paper.
