# Supplementary material for: Avenues of decarbonisation in the dynamics of processed food supply chains: Towards responsible production consumption
Source: Heliyon. 2024 Feb 17;10(5):e26456. doi: 10.1016/j.heliyon.2024.e26456 (PMC10918021; doi:10.1016/j.heliyon.2024.e26456)
Supplement: Multimedia component 1 [file mmc1.docx]

# SURVEY QUESTIONNAIRE

**Subject:** Survey questionnaire to assess the effect of various dimensions associated with the decarbonization of the processed food supply chain performance system.

As a part of doctorate degree research work being conducted jointly at the Department of Industrial and Production Engineering, National Institute of Technology Jalandhar and Indian Institute of Technology Mandi (India). Myself Janpriy Sharma (Research Scholar) conducting a survey for assessment of the food supply chain performance systems under different contexts. Your feedback in this regard will provide me a significant boost to achieve my research objectives. The objective of this survey is purely study and academic based; therefore, all responses will be kept strictly confidential. For more enquiries you may contact supervisor Dr. Mohit Tyagi ([tyagim@nitj.ac.in](mailto:tyagim@nitj.ac.in)) and Dr Satvasheel Powar ([satvasheel@iitmandi.ac.in](mailto:satvasheel@iitmandi.ac.in)).

For the same, I shall be very grateful to you.

## With Regards


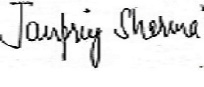


Janpriy Sharma Research Scholar

Department of Industrial and Production Engineering Dr B R Ambedkar National Institute of Technology Jalandhar – 144011

Enclosure:

Part 1: Company Detail

Part 2: Questionnaire

**Part 1- Company Profile**

**Name of the Company** ………………………………………..

1. **Type of company**

A. [ ] Processing B. [ ] Packaging

C. [ ] Logistic Provider D. [ ] Distribution and Retailing

1. **Establishment of the company**

[ ] Before Year 2005

[ ] 2005 to 2008

[ ] 2008 to 2011

[ ] After 2011

1. **Please indicate the number of employees at your company**

A. [ ] Less than 100 B. [ ] Between 100 to 500

C. [ ] Between 500 to 1000 D. [ ] More than 1000

1. **Please indicate the Annual Turnover of the recent three years** 2020 : ………………………………

2021 : ………………………………

2022 : ………………………………

1. **Respondent’s current designation in the company**………………………………………………
2. **Working experience in the field of food processing and supply chain** (In Years)………………
3. **Respondent’s Email ID**……………………………………………………………………………

**Part 2: Questionnaire**

| **Kindly rate, sustainability practices affecting food supply chain performance system** | | **1** | **2** | **3** | **4** | **5** |
| --- | --- | --- | --- | --- | --- | --- |
|  |  | **Completely Disagree** | **Somewhat Disagree** | **Cannot Decide** | **Somewhat Agree** | **Strongly Agree** |
| **Procurement initiatives** | Baselining the operational procedurals and frameworks governing sustainability |  |  |  |  |  |
|  | Incitation of ISO 20400 protocols. |  |  |  |  |  |
|  | Fostering partnerships with firms promoting sustainability and innovation. |  |  |  |  |  |
|  | Circular procurement practices. |  |  |  |  |  |
| **Sustainable manufacturing practices** | Opting technologies and procedures emphasizing cleaner production. |  |  |  |  |  |
|  | Minimized wastage during production stages. |  |  |  |  |  |
|  | Operations favoring economic viability. |  |  |  |  |  |
| **Research and development initiatives** | Rendering necessary technical support and expertise. |  |  |  |  |  |
|  | An innovative solution to promote sustainable practices. |  |  |  |  |  |
| **Distribution initiatives** | Low carbon distribution logistics practices. |  |  |  |  |  |
|  | Traceability and monitoring of the food items. |  |  |  |  |  |
| **Customer-based** | Preferences for the product binding sustainability. |  |  |  |  |  |
|  | Opting products conforming to quality standards. |  |  |  |  |  |
| **External factors** | Government rules and regulations evoking sustainability in FSC operation. |  |  |  |  |  |
|  | Corporate social responsibility. |  |  |  |  |  |
|  | Promotion of sustainable consumption. |  |  |  |  |  |
|  | Better end of product life. |  |  |  |  |  |

*Your comments related to enactors of decarbonization of food supply chain (If, any)*

*____________________________________________________________________________________*
